# Multi-drug pharmacotyping improves therapy prediction in pancreatic cancer organoids

**DOI:** 10.1186/s12935-025-03969-7

**Published:** 2025-09-13

**Authors:** Katharina Wansch, Uwe Pelzer, François Schneider, Florian Dölvers, Anna Kühn, Mihnea P. Dragomir, Jana Ihlow, Georg Hilfenhaus, Loredana Vecchione, Matthäus Felsenstein, Dou Ma, Markus Lerchbaumer, Christian Jürgensen, Marcus Bahra, Adrian E. Granada, Gregor Duwe, Sebastian Stintzing, Ulrich Keilholz, Christopher C. M. Neumann

**Affiliations:** 1 Department of Hematology, Oncology and Tumor Immunology, Charité-Universitätsmedizin Berlin, Freie Universität Berlin, Humboldt-Universität zu Berlin, Berlin Institute of Health, Berlin, Germany; 2 Department of Pathology, Charité-Universitätsmedizin Berlin, Freie Universität Berlin, Humboldt-Universität zu Berlin and Berlin Institute of Health, Berlin, Germany; 3 German Cancer Consortium (DKTK), Partner Site Berlin and German Cancer Research Center (DKFZ), Heidelberg, Germany; 4 Berlin Institute of Health (BIH), Berlin, Germany; 5 Department of Surgery|CCM|CVK, Charité-Universitätsmedizin Berlin, Freie Universität Berlin, Humboldt-Universität zu Berlin and Berlin Institute of Health, Berlin, Germany; 6 Department of Radiology, Charité-Universitätsmedizin Berlin, Freie Universität Berlin, Humboldt-Universität zu Berlin and Berlin Institute of Health, Berlin, Germany; 7 Department of Hepatology and Gastroenterology, Charité-Universitätsmedizin Berlin, Freie Universität Berlin, Humboldt-Universität zu Berlin and Berlin Institute of Health, Berlin, Germany; 8 Department of Surgical Oncology and Robotics, Krankenhaus Waldfriede, Lehrkrankenhaus der Charité, Berlin, Germany; 9 Charité Comprehensive Cancer Center, Charité – Universitätsmedizin Berlin, Berlin, Germany; 10 Department of Urology and Pediatric Urology, University Medical Center Johannes Gutenberg University, Mainz, Germany

**Keywords:** Pancreatic adenocarcinoma, Patient-derived organoids, Multi-Drug response metrics, Pharmacokinetic modelling

## Abstract

Patient-Derived Organoids (PDOs) represent a promising technology for therapy prediction in pancreatic cancer, with the potential of enhancing treatment outcomes and allowing more effective, personalized treatment choices. However, classification approaches into sensitive and resistant models remain very variable and are based on single-agent testing only, neglecting interactive effects of multi-drug combinations. Here, we established 13 PDOs and performed both single-agent and multi-drug testing. By comparing different clustering approaches of drug-response metrics and establishing a new classification approach based on pharmacokinetic modelling, we were able to evaluate which score best predicts the clinical response of patients. Our newly developed score considered the Area Under The Curve (AUC) of cell viability curves and reached a prediction accuracy of 85%. Our data supports previous findings for PDOs to constitute an effective platform for translational drug testing. Furthermore, our results suggest that the AUC is a more accurate drug-response metric than the half maximal inhibitory concentration (IC_50_), and that multi-drug testing yields a higher accuracy than single-agent testing. The methodology and outcomes presented in this study are of critical relevance for future PDO-based translational trials as they allow a new physiology-based approach towards multi-drug testing and classification of organoid response, which improves PDO prediction accuracy.

**Supplementary Information:**

The online version contains supplementary material available at 10.1186/s12935-025-03969-7.

## Introduction

Pancreatic ductal adenocarcinoma (PDAC) is one of the deadliest types of cancer, with a 5-year survival rate of 11% only [1]. Moreover, PDAC is expected to be the second most common cause of cancer-related death in the world by 2030 [1–3]. The poor prognosis of PDAC can be attributed to unspecific symptoms leading to late diagnosis as well as early metastasis and high resistance towards chemotherapeutic agents. Surgical resection represents the only curative treatment option at a local disease stage, which is possible for 20% of all patients. Once resected, the 5-year survival rate is reported to be 20% [4, 5], while 80% of patients relapse after surgery and adjuvant chemotherapy. The majority of patients are diagnosed at a more advanced disease stage. For patients with borderline resectable or locally advanced PDAC, neoadjuvant chemotherapy can achieve secondary resectability with a chance of 40% [6]. Patients with metastasized or inoperable disease are treated with palliative chemotherapy. Chemotherapy is therefore an integral component of the treatment of all stages of PDAC as it is used both perioperatively and in a palliative setting. Unfortunately, most patients do not show long-term response to chemotherapy which underlines the high levels of chemotherapeutic resistance and high failure rates in the multi-modal treatment for PDAC [7].

While individualized therapies have emerged in lung- or colorectal cancer [8–10], the driver mutations in PDAC, including *KRAS* (apart from rare *KRAS*^G12C^), *CDKN2A*, *TP53* and *SMAD4* cannot be therapeutically targeted at the moment [11, 12]. At this point, conventional chemotherapy remains the treatment option in clinical practice. Three different chemotherapeutic regimens are currently used: mFOLFIRINOX (Folic acid, 5-Fluorouracil, Irinotecan and Oxaliplatin) [13], Gemcitabine combined with nab-Paclitaxel [14] and Gemcitabine monotherapy [15]. So far, there a no clinically established biomarkers to predict which patient will respond to which chemotherapy and treatment choices are mostly based on patients’ age and performance status [16]. To predict patients’ response and enable more informed treatment choices, the field of Patient Derived Organoids (PDOs) has gained attention within the last years. PDOs are three-dimensional in-vitro models of epithelial cells grown in an extracellular matrix. Due to their 3D structure, PDO models exhibit a tumor biology more closely resembling the original tumor than 2D cell cultures. This was previously confirmed in different cancer entities on histological, genomic and transcriptomic levels [17–21]. PDO models also display more heterogenous drug responses than monolayer cell cultures [22–24] and are thought to more closely represent drug responses obtained by in-vivo models [22, 25, 26]. In contrast, PDX animal models also take into account the tumor microenvironment depicting tumor vascularization, tumor stroma and immunological interactions [27–30]. However, despite these advantages, PDX in-vivo models are costly and do not permit pharmacotyping of a broad range of substances in a time-efficient manner, which is required for personalized decision-making in the clinical setting. Because of these various practical and biological advantages, PDOs have become a versatile tool for studying PDAC [31–33]. PDOs consisting purely of epithelial cells, however, are a limited representation of the original tumor as they lack the tumor microenvironment and cannot be used to study interactions of tumor cells with stromal and immune cells [34]. Despite these limitations, growing evidence suggests a relevant predictive power of PDOs for clinical patients’ response [35–39]. Although individual groups are achieving promising results, there is a lack of standardized methods for the evaluation of drug response in PDOs, resulting in highly variable approaches towards defining drug response. Furthermore, despite patients being administered several drugs at the same time with the drugs giving rise to important pharmacodynamic interactions, previous studies only assessed single agent testing on PDO models [40–44]. Since PDO models have great potential to act as dynamic predictive biomarkers, these models are implemented in ongoing and future clinical trials [45]. For future clinical trials to be conducted, however, a reliable way of classifying models into resistant and sensitive should be standardized across the field of PDO-based translational research. The variability of response classification as well as the lack of multi-drug testing in PDAC PDOs represent two major limitations on the way to implementing PDOs in clinical trials.

In the work presented, these limitations were addressed. We evaluate different scores to predict patients’ response and integrate testing chemotherapeutics both as single agents and in combination. This way, clinical conditions were replicated more closely. Moreover, we established statistical clustering and pharmacokinetic models to compare and challenge current definitions of in-vitro PDO response. PDOs were successfully pharmacotyped towards single chemotherapeutic agents as well as the multi-drug therapy of mFOLFIRINOX and gemcitabine/paclitaxel and results of in-vitro drug testing were compared to the clinical response of patients in 13 cases. Our results suggest that multi-agent testing based on physiological pharmacokinetic models is feasible and achieves higher accuracies than single-agent testing, making it the ideal method for future PDO-based, multi-agent clinical trials.

## Materials and methods

### Patient selection and ethics statement

**Fig. 1:**
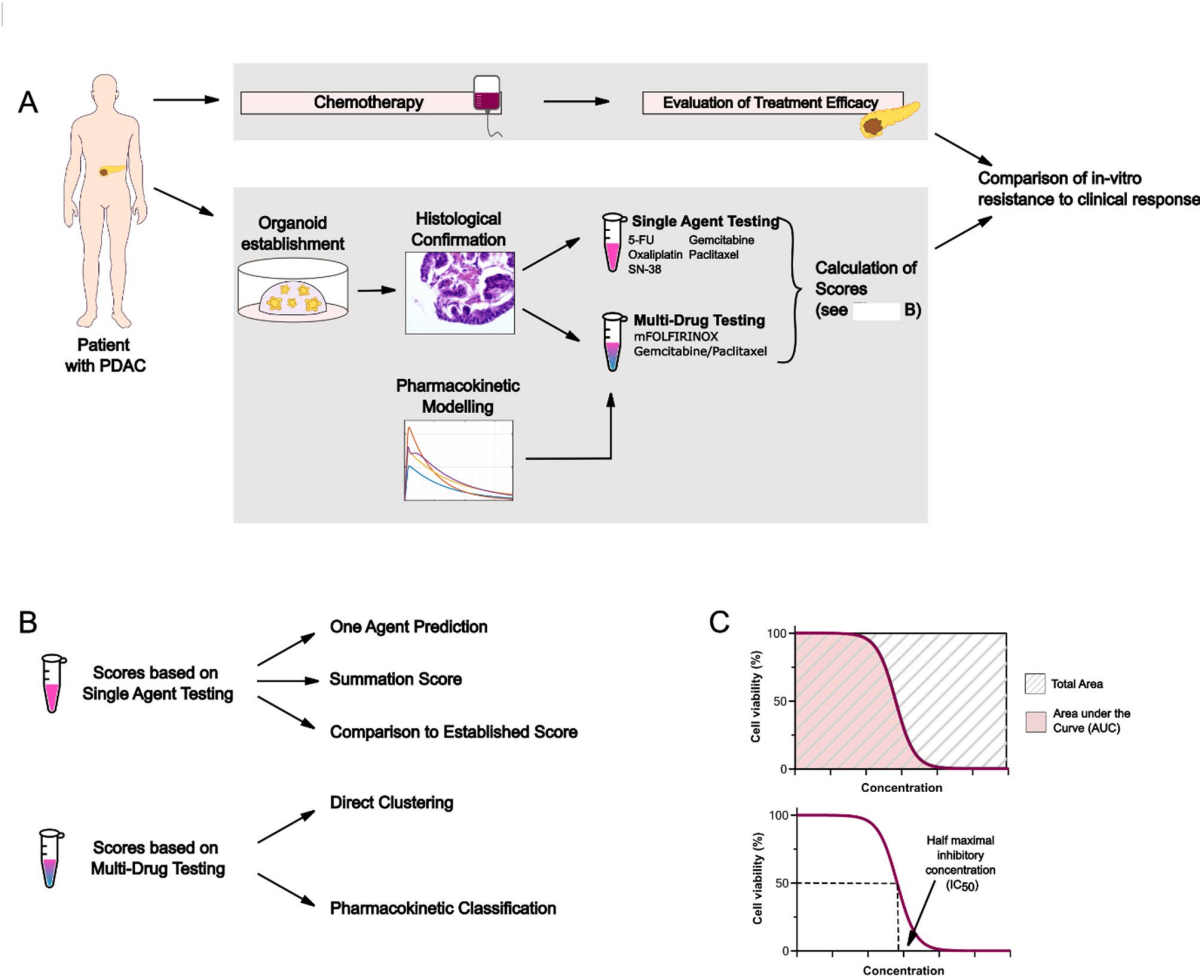
Workflow for determining in-vitro response and comparing in-vitro results to clinical treatment response. **A** Samples were derived from primary tumors and metastases. While patients were undergoing chemotherapy, organoids were established and malignancy was histologically confirmed. Organoids were then treated with single agents and multi-drug therapy based on pharmacokinetic modelling. Different scores were calculated and in-vitro results were compared to the clinical outcome for each patient. **B** Organoids were exposed to single agent and multi-drug treatment. For single agent testing, three different methods were used to classify drug response: prediction based on one single agent, a summation score and, for comparison, an established score based on previous work from Beutel et al. For multi-drug testing, a direct clustering and a pharmacokinetic approach were used to classify PDOs as sensitive or resistant. **C** Drug response metrics AUC and IC_50_ were used for all scores. To calculate the summation score, the AUC was normalized to the total area for each drug. The log[IC_50_] was normalized to the highest concentration used for the in-vitro drug test

For this study, tumor samples were obtained from treatment-naïve patients suffering from PDAC and were consequently treated at the Charité-Universitätsmedizin Berlin and at the Waldfriede Hospital Berlin-Zehlendorf between November 2021 and May 2023. The tissue extraction as well as all further steps were performed in accordance with the ethics approval (EA1/157/21) and signed consent was obtained from all patients. Samples were only included for pharmacotyping if PDAC was histopathologically confirmed and clinical follow-up was known (see Fig. 1A). Patients had to receive at least two cycles of chemotherapy to be included in this study. Maximum follow-up for our patient cohort was 18 months.

### Assessment of treatment efficacy

For resected patients, therapy response was clinically evaluated based on radiological imaging after 6 months. Patients were classified as resistant when suffering from a relapse within 6 months after the start of adjuvant chemotherapy. If no relapse occurred, they were classified as sensitive. For sub-analyses of clinical response, the Time To Relapse (TTR) was identified for patients with resectable disease. This was defined as the time from the date of surgery to the date of tumor recurrence.

Treatment response of metastatic patients was evaluated based on RECIST 1.1 criteria [46] of the first CT-staging after a minimum of three months of therapy. Metastatic PDACs were classified as sensitive when partial response or stable disease was detected. When tumors progressed under therapy, they were classified as resistant. Moreover, the decrease in CA 19 − 9 upon the first two to four months of palliative first-line therapy was used as an additional parameter for therapy response. For further sub-analyses, resected patients with a TTR shorter than 6 months or metastatic patients with a decrease in CA 19 − 9 of less than 60% were classified as resistant.

### Isolation and establishment of patient-derived organoids

Organoids were established from the primary tumor via surgical specimens and from metastatic sides via liver biopsies. Samples were manually cut into small pieces of about 1 mm and digested at 37 °C in a solution containing 100 µg/ml DNAse I (VWR, Radnor, PA, USA), 100 µg/ml Dispase (STEMCELL Technologies, Vancouver, Canada), 125 µg/ml Collagenase II (Sigma-Aldrich, Merck, Darmstadt, Germany), 1:2000 Rock-Inhibitor (Abmole Bioscience, Houston, TX, USA) and 1:200 Amphotericin B (Sigma-Aldrich, Merck, Darmstadt, Germany). Biopsy specimens were enzymatically dissociated for 5 to 30 min, while tumor specimens were incubated for 2 to 3 h. After dissociation, the cells were filtered using a using a sterile 100 μm filter. Remaining cell clumps were discarded. After performing red blood cell lysis (Miltenyi Biotec, Bergisch-Gladbach, Germany), cells were plated in Cultrex Reduced Growth Factor BME, Type 2 (R&D Systems, Minneapolis, MN, USA) at a density of 50.000 cells per 30 µl dome. Domes were incubated for 30 min at 37 °C to solidify. Afterwards, human pancreas expansion and isolation medium as established by Broutier et al. [47] was added (see Supplementary Table 1). To prevent contamination, Amphotericin B was added at a dilution of 1:200 for the first week and Mycoplasma tests were regularly performed using the Mycoplasma detection kit (Applied biological Materials, Richmond, Canada). Growth medium was exchanged every two to three days. When a size of more than 200 μm or a density of 70% was reached, organoids were split mechanically and enzymatically using TrypLE Express (Thermo Fisher Scientific, Waltham, MA, USA) and plated in Cultrex at a ratio of 1:2. The first passage was reached within a median of 14 days and further splitting was usually necessary once a week.

### Histology and immunohistochemistry

Samples were embedded for histological analysis between passages 4 and 15. For embedding, Cultrex was dissolved by adding 500 µl PFA (4% in PBS) to PDOs at 4 °C for one hour. PDOs were detached from the plate, washed with PBS and resuspended in histogel (Thermo Fisher Scientific, Waltham, MA, USA). Further steps were performed at the Charité Institute of Pathology. After paraffin-embedding, blocks were cut into 3 μm sections. H&E-staining was carried out with the Tissue Tek Prisma Plus Automated Slide Stainer (SAKURA). Immunohistochemical staining was carried out with the BenchMark XT immunostainer (Ventana Medical Systems, Tucson, AZ). The following antibodies were used: anti-Ki-67 (MIB-1, Dako, 1:50), anti-Vimentin (V9, Dako, 1:5000), anti-CA 19 − 9 (1116-NS-19-9, Dako, 1:500), anti-CK 19 (RCK108, BioGenex, 1:100), anti-SMAD4 (EP618Y, abcam, 1:200), anti-p53 antibody (DO-7, Dako, 1:50), and anti-GATA6 antibody (Q92908, R&D Systems, 1:100). Slides were digitalized and analyzed by a reference pathologist.

### PDO pharmacotyping

PDO drug response was tested in technical triplicates between passages 5 and 15. PDOs were collected and digested to a single-cell suspension by TrypLE (Thermo Fisher Scientific, Waltham, MA, USA). Cells were counted, the proportion of single cells and cell viability was measured using Acridine Orange/Propium Iodide stain (BioCat, Heidelberg, Germany) with the LUNA automated cell counter (BioCat, Heidelberg, Germany). For cell viability assays to be conducted, the proportion of single cells needed to be at least 90%. Having assured to be within the linear range of the CellTiter-Glo^®^ Luminescent Cell Viability Assay (Promega, Walldorf, Germany), 2,000 cells were plated into each well of a 96 Well-plate. 200 µl medium containing 10 µM Rock Inhibitor were added after solidification of the domes. PDOs were fully formed by day 3 after plating. After 4 days of incubation, 100 µl of medium were discarded manually using a multi-channel pipette and drugs were added in seven concentrations. For single agents, a 10-fold dilution series was used. The maximum concentrations were 1 mM for 5-FU, 100 µM for Oxaliplatin, 50 µM for Gemcitabine and 10 µM for SN-38 and Paclitaxel. Multi-Drug therapies were added in a 4-fold dilution series, ranging from 64 x c_max/tissue_ to 1/64 x c_max/tissue_, where c_max/tissue_ was defined as the pharmacokinetically calculated tissue concentration of each drug (see Sect. 2.6). These concentration ranges were based on previously performed PDAC PDO pharmacotyping studies to ensure comparability [20, 35, 48, 49]. All cytostatics were purchased from Selleckchem and calcium folinate was purchased from Biozol Diagnostica. Cell viability was measured using the CellTiter-Glo^®^ Luminescent Cell Viability Assay after 3 days of treatment. 100 µl of medium were discarded and 100 µl of reagent were added using a multi-channel pipette. After 30 min of incubation, luminescence was measured using the VICTOR^®^ Nivo™ multiplate reader (Revvity Inc., Waltham, MA, USA). Outliers in luminescence measurements were identified using the ROUT method in GraphPad Prism (Version 10.2.3). Values were excluded when the Q-value exceeded 0.01. AUC, IC_50_ and log[IC_50_] were subsequently calculated in GraphPad Prism based on normalized cell viability values. To calculate IC_50_ and log[IC_50_], the four parameter log-logistic function Y = 100/(1+(IC_50_/X)^HillSlope) was used. AUC-values were calculated using the trapezoidal method and the total area was used for further analyses. Dose-response curves for single agents and multi-drug testing and violin plots of AUC- and log[IC_50_]-values were created using GraphPad Prism (Version 10.2.3).

### Pharmacokinetic modelling

To estimate tissue concentrations of chemotherapeutic agents, mathematical pharmacokinetic models were implemented based on pharmacokinetic values previously reported in the literature [50–70]. In these simulations, the dose and infusion time of each chemotherapeutic agent, the rate of distribution into the tissue as well as the rate of distribution back into the bloodstream were considered. Furthermore, metabolization and elimination of the chemotherapeutic agent were taken into account. Pharmacokinetic modelling was based on one-, two- and three-compartment modelling as described by Schiffter-Weinle [71]. Within these models, the body was viewed as a system of different compartments, with a distinction made between the central and the peripheral compartments. When modelling, chemotherapeutic agents were infused into the central compartment (blood) and distributed to the peripheral compartments (tissue) by linear and/or saturable kinetics. Saturation kinetics were used in transport processes that were reported to be dependent on enzymes or proteins. In these cases, Michaelis-Menten kinetics were applied. The number of compartments used in each model depended on the drug’s distribution patterns previously reported in the literature [50–70].

One-compartment models were used for drugs that distributed in negligible time within the entire body and were known to have similar kinetics in all body areas (blood and tissue). In contrast, models with two or more compartments were implemented for drugs that were previously reported to distribute heterogeneously within the body. The blood stream always represented the central compartment. Whenever more than one peripheral compartment was employed, the first peripheral compartment was defined to be the one within which the drug was distributed most rapidly (e.g. GI-tract). The second peripheral compartment represented tissues with a lower transfer rate (e.g. fatty tissue). The intercompartmental clearance rates described the speed of drug distribution and the elimination rate described the speed of drug excretion. In the case of Michaelis-Menten kinetics, this depended on the maximum transport velocity and the Michaelis-Menten constant. Tissue concentrations were deduced from the peripheral compartment. When using three-compartment models, the concentration depicting the tumor concentration was calculated in the first peripheral compartment. Extracting tissue concentrations from the first peripheral compartment was based on blood flow rates measured previously in CT-perfusion studies of PDAC patients [72]. To identify diffusion rates and the number of compartments of each chemotherapeutic agent, a systematic literature review was conducted on PubMed. Taking into account that PDAC occurs mainly in elderly patients [73], studies with pediatric patient populations were excluded. All calculations were done in MatLab/Simulink (R2022b).

For one compartment models, the following equation was used to calculate the change in the amount of drug in the body over time, where *x*_*1*_ *[mg]* represented the amount of drug in the central compartment, *D [mg]* the dose of the drug, *T [h]* the infusion time and *e [mg]* the amount of drug excreted (see Supplementary Table 2):

$$\:\frac{d{x}_{1}}{dt}=\frac{D}{T}-\frac{d\text{e}}{dt}$$

Excretion of the drug was described by linear kinetics, where *Cl [l/h]* represented the clearance rate and *V*_*1*_ *[l]* the volume of distribution of the central compartment:

$$\:\frac{d\text{e}}{dt}=\frac{Cl}{{V}_{1}}\cdot\:{x}_{1}$$

For two-compartment models, the change in the amount of drug in the central compartment over time was described as

$$\:\frac{d{x}_{1}}{dt}=\frac{D}{T}-\frac{{Q}_{1}}{{V}_{1}}\cdot\:{x}_{1}+\frac{{Q}_{1}}{{V}_{2}}\cdot\:{x}_{2}-\frac{d\text{e}}{dt}$$

with *Q*_*1*_ *[l/h]* being the intercompartmental clearance rate between central and peripheral compartment, *x*_*2*_ *[mg]* the amount of drug in the peripheral compartment and *V*_*2*_ *[l]* the peripheral volume of distribution.

Elimination was either linear as described above or saturable following Michaelis-Menten kinetics, where *v*_*max*_ *[mg/h]* equals the maximum speed of excretion in and *K*_*m*_ *[mg/l]* is the Michaelis-Menten constant.

$$\:\frac{d\text{e}}{dt}=\frac{{v}_{\text{m}\text{a}\text{x}}\cdot\:{c}_{1}}{{K}_{m}+{c}_{1}}$$

The change in the amount of drug in the peripheral compartment, x_2_ *[mg]*, over time was calculated as follows:

$$\:\frac{d{x}_{2}}{dt}=\frac{{Q}_{1}}{{V}_{1}}\cdot\:{x}_{1}-\frac{{Q}_{1}}{{V}_{2}}\cdot\:{x}_{2}$$

For three-compartment models, the same equations applied for both the elimination of the drug and the amount of drug in the first peripheral compartment. The change in the amount of drug in the central compartment over time was calculated as follows:

$$\begin{aligned}\:\frac{d{x}_{1}}{dt}&=\frac{D}{T}-\frac{{Q}_{1}}{{V}_{1}}\cdot\:{x}_{1}+\frac{{Q}_{1}}{{V}_{2}}\cdot\:{x}_{2}\\&\quad-\frac{{Q}_{2}}{{V}_{1}}\cdot\:{x}_{1}+\frac{{Q}_{2}}{{V}_{3}}\cdot\:{x}_{3}-\frac{d\text{e}}{dt}\end{aligned}$$

where *Q*_*2*_ *[l/h]* described the intercompartmental clearance rate between central and second peripheral compartment and *V*_*3*_ *[l]* described the volume of distribution of the second peripheral compartment.

### Response classification

Cell viabiliy curves obtained from single agent and multi-drug testing as described in Sect. 2.5 were further analyzed with respect to the AUC and log[IC_50_] using statistical clustering methods and pharmacokinetic classifications (see Fig. 1B). For Clustering of drug-response metrics, we chose a partition-based clustering approach with a pre-defined number of 3 classes (corresponding to low, intermediary and high sensitivity). The Jenks Natural Breaks (JNB) Classification was chosen as this approach minimizes variance within a group while maximizing variance between groups. Moreover, it was previously used by several other groups to cluster AUC-values [25, 35, 74, 75]. The number of iterations was not pre-specified, but a minimum goodness of fit of 90% had to be achieved for each dataset. These analyses were conducted in Microsoft Excel and R/R-Studio (Version 4.3.2).

#### Prediction accuracy of using one single agent

First, the prediction accuracy was assessed using single agent testing. This way the clinical response of patients who had received multiple chemotherapeutics in the clinic (mFOLFIRINOX or gemcitabine/paclitaxel), was linked to results of one single agent only. AUC- and log[IC_50_]-values of single agent testing were grouped using the JNB classification into high, intermediate and low responders and were then compared to the clinical response of patients.

#### Summation score of single agent testing

For the in-vitro results of single agent testing to be matched with the multi-drug treatment of patients in the clinic, in-vitro results of single agent tests had to be combined. This was implemented by adding normalized AUC (nAUC) and normalized log[IC_50_]-values (nlog[IC_50_]) from single drug testing. AUC-values were normalized with respect to the total area accounting for 100% of untreated live cells at the highest concentration examined (see Fig. 1C). For mFOLFIRINOX, the nAUC-values of oxaliplatin, 5-FU and SN-38 were added. For gemcitabine/paclitaxel (Gem/Pac), the nAUC of gemcitabine and paclitaxel were added. log[IC_50_]*-*values were normalized with respect to the maximum concentration used for each drug. The same summation score was calculated for nlog[IC_50_]*-*values for both regimens (see Table 1). JNB classification was used to group PDOs into sensitive, intermediate and resistant based on the sums of nAUC- and nlog[IC_50_]*-*values described above.

**Table 1:** Calculation of summation scores for gem/pac and mFOLFIRINOX

Summation Score	Calculation
nAUC(mFOLFIRINOX)	nAUC(oxaliplatin) + nAUC(5-FU) + nAUC(SN-38)
nAUC(Gem/Pac)	nAUC(gemcitabine) + nAUC(paclitaxel)
nlog[IC_50_] (mFOLFIRINOX)	nlog[IC_50_] (oxaliplatin) + nlog[IC_50_] (5-FU) + nlog[IC_50_] (SN-38)
nlog[IC_50_] (Gem/Pac)	nlog[IC_50_] (gemcitabine) + nlog[IC_50_] (paclitaxel)

#### Direct clustering using multi-drug testing

In in-vitro multi-drug tests, PDOs are directly exposed to combinations of chemotherapeutics. Thus, no scoring system, as for adding single-agent testing, was required. For mFOLFIRINOX, PDOs were treated with folic acid, 5-FU, irinotecan and oxaliplatin at the same time. For gemcitabine/paclitaxel, PDOs were simulatenously treated with gemcitabine and paclitaxel. JNB classification was used to classify PDOs as sensitive, intermediate and resistant based on AUC- and log[IC_50_]*-*values for mFOLFIRINOX and gemcitabine/paclitaxel.

#### Comparison to established statistical score

For a comparison to previously published scores to be made, the data collected in this work was directly compared to the score first published by Beutel et al. [35]. In short, JNB classification was used to classify AUC-values of single agents into Low, intermediate and high responders. Low-responders were assigned a numerical value of 3, intermediate-responders a value of 2 and high-responders were assigned a value of 1. When patients were treated with multiple chemotherapeutics at the same time (mFOLFIRINOX or gemcitabine/paclitaxel) the mean of the added values was calculated. PDOs were classified as resistant when the mean value was greater than 2.

#### Pharmacokinetic classification

In a pharmacokinetic approach, IC_50_-values of mFOLFIRINOX, gemcitabine/paclitaxel and gemcitabine were compared to the maximum tissue concentrations (c_max/tissue_) of all drugs used in the individual regimens calculated in Sect. 2.6.2. PDOs were classified as sensitive if the IC_50_ was less than c_max/tissue_ and as resistant if it was higher than c_max/tissue_.

#### Comparison to clinical response

All scores were compared to patients’ clinical response and the accuracy for each score was calculated seperately for first- and second-line therapies. When PDOs were defined to be sensitive or intermediate to a chemotherapeutic drug, the in-vitro result was classified as sensitive.

#### Uncertainty Estimation

To estimate the uncertainty of each classification method, leave-one-out cross validation and bootstrapping were performed in R/R-Studio (Version 4.3.2). For leave-one-out cross validation, one PDO model was excluded from the data set which was used to train the classification approach in analogy to Ooft et al. [76]. Afterwards, the classification was applied on this PDO model and the result was compared to the clinic of the corresponding patient. This was repeated for all 13 PDO models. Bootstrapping was implemented according to Davison et al. [77] and performed with 2,000 iterations using the boot-package [77, 78]. Confidence Intervals were calculated using the BCa (Bias-corrected and accelerated) method [78].

### Classifying in-vitro parameters from in-vivo therapy response

In a further step, a reverse approach was considered. This was implemented in analagoy Ooft et al. [76]. In short, the drug concentration that resulted in the highest variability in cell viabilty across models was identified. This was done by calculating the standard deviation at each dose. Next, cell viabilities at this concentration were compared to the clinical outcome. We also compared AUC-values to the clinical outcome. Since AUC-values represented the integral of the entire concentration range, no variance needed to be determined. ROC-curves were calculated in GraphPad-Prism (Version 10.2.3) for both cell viability and AUC-values. Based on the ROC-curve, the optimal threshold to separate sensitive vs. resistant samples was identified and sensitivity and specificity of both metrics were determined.

### Assessment of pharmacodynamic interactions in multi-drug testing

We assessed whether chemotherapy regimens were synergistic, antagonistic or additive in our PDO models. First, we calculated the expected effect of each combination (E_mFOLFIRINOX_ and E_Gem/Pac_) based on the Bliss-independence model [79]. This was done by calculating the reduction in cell viability at the c_max/tissue_ for each single agent (E_single agent_). The expected effect of the regimen was then identified using the following formula:

$$\begin{aligned}{E}_{mFOLFIRINOX}&={E}_{5-FU}+{E}_{Ox}+{E}_{SN-38}\\&\quad-({E}_{5-FU}\times\:{E}_{Ox})\\&\quad-({E}_{5-FU}\times\:{E}_{SN-38})-\:\end{aligned}$$

$$\:{(E}_{Ox}\times\:{E}_{SN-38})+({E}_{5-FU}\times\:{E}_{Ox}\times\:{E}_{SN-38})$$

$$\:{E}_{Gem/Pac}={E}_{Gem}+{E}_{Pac}-\left({E}_{Gem}\times\:{E}_{Pac}\right)$$

Next, the expected effect was compared to the observed effect of each multi-drug chemotherapy at c_max/tissue_ in-vitro. If the observed effect exceeded the expected effect, the regimen was classified as synergistic. If the observed effect was lower than the expected effect, it was classified as antagonistic. If both effects were equal, the combination was classified as additive. The magnitude of the interaction was quantified by subtracting the expected effect from the observed effect. This was done for each patient and each multi-drug therapy individually.

## Results

### Successful PDO establishment in 58% of patients

In total, 43 patients were included for this study and PDO establishment was successful in 25 patients (58%). Samples were taken from treatment-naïve PDAC patients. All patients were treated and clinically followed up at between November 2021 and May 2024 at the University Hospital Charité – Universitätsmedizin Berlin and the Hospital Waldfriede Berlin-Zehlendorf. 17 patients received adjuvant chemotherapy, 13 of whom could be followed-up clinically for at least 6 months. These 13 patients were included for subsequent analyses and the development of our pharmacotyping classification.

Regarding establishment rates, we did not see a significant difference between successful PDO growth and failed PDO establishment regarding sex, age and UICC stage of patients (see Supplementary Table 3). Samples from metastases were established with a success rate of 89% compared with 50% for samples derived from primary tumors (*p* = 0.085). There were no significant differences in PDO establishment efficacy regarding sample size and weight, T-Stage, N-Stage and histological grading.

It took an average of 13 days until PDOs could first be passaged and an average of 29 days until PDOs reached passage three and were considered as established. Pharmacotyping was successful in all 13 patients with a mean coefficient of variance between technical replicates of 14.67%. A total of two drug tests were repeated, once because of a pipetting error and once because of low cell viability of the control.

### Histopathological assessment confirms malignancy in all PDO models

**Fig. 2:**
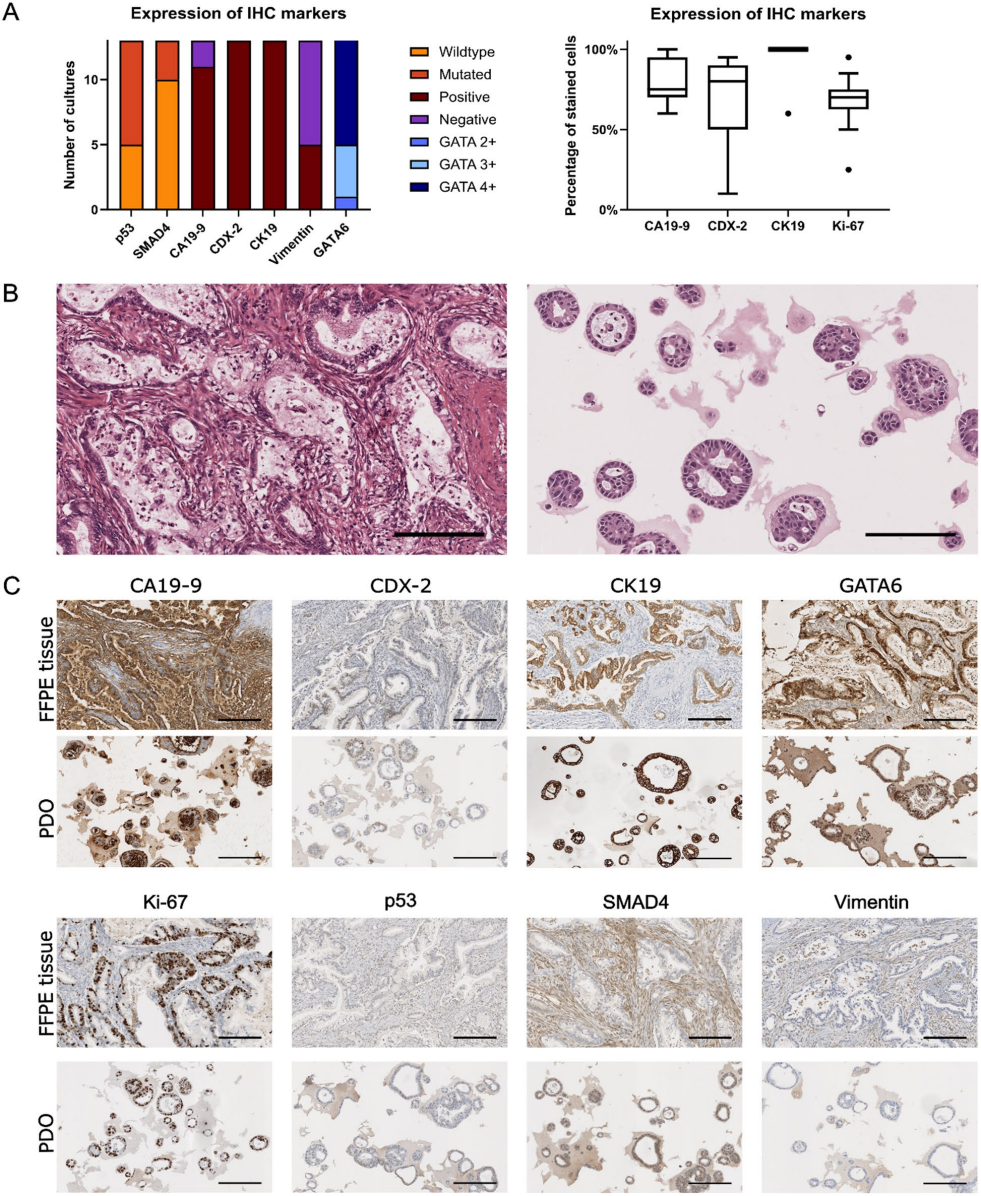
HE and immunohistochemical staining of FFPE tissue and PDO model from one representative patient. Scale bars represent 200 μm. **A**. Bar graphs for p53, SMAD4, CA 19 − 9, CDX-2, CK19, Vimentin and GATA6 are shown on the left, revealing that expression of markers is variable across cultures, with all cultures expressing CDX-2 and CK19. On the right, the strength of expression (number of cells stained) in positive cultures is shown for CA 19 − 9, CDX-2, CK19 and Ki-67. The mean percentage of stained cells is 81% for CA 19 − 9, 70% for CDX-2 and 96% for CK19. The mean proliferation index in PDO models is 68%, ranging from 25–95%. **B** Both the FFPE tissue and the PDO show a similar gland-forming morphology with cell debris in the lumen. The cells show enlarged nuclei and medium grade nuclear pleomorphism. **C** Immunohistochemical staining reveals similarity between PDO models and the corresponding FFPE tissue. Both FFPE tissue and PDOs are positive for the tumor marker CA 19 − 9, CK19 and GATA6. Ki-67 expression is increased to 70% in the organoid model compared to 60% in the FFPE tissue

Malignancy was confirmed by histopathological evaluation based on morphology and expression of immunohistochemical markers. Both organoid morphology in H&E staining as well as immunohistochemical stainings (CA 19 − 9, CDX-2, CK19, GATA6, ki-67, p53, SMAD4, Vimentin) showed high similarity of PDO models and corresponding FFPE tissue (see Fig. 2B and C). P53 was mutated in 8 of 13 models and SMAD4 was mutated in 3 of 13 models. Ki-67 expression ranged between 25% and 95% with a mean Ki-67-expression of 68%. All cultures were positive for CDX-2 and CK19, 11 of 13 cultures expressed CA 19 − 9. Vimentin was focal positive in 5 cultures (see Fig. 2A).

### Patient characteristics

Eight of thirteen patients included in this study were diagnosed at a resectable stage (see Table 2). All eight samples were obtained from surgical resections. R0-resection was achieved in six out of eight patients (6/8), while two patients (2/8) had an R1-resection status. Five samples were obtained at a metastasized stage. The samples collected from metastasized patients were established from liver metastases (4/5) and from the primary tumor (1/5). Samples were collected during palliative surgeries or punch biopsies. The mean age of the patient cohort was 68 years (+/- 10 years), similarly aligned in resected and metastatic groups. On average, 5.5 weeks passed between sample collection and therapy initiation. Therapy started earlier in metastasized patients than in patients who underwent surgery with curative intention (2.8 vs. 7.1 weeks). Seven patients received mFOLFIRINOX as a first-line therapy. Two patients received gemcitabine/nab-paclitaxel and four patients were treated with gemcitabine monotherapy. Six patients showed initial response to mFOLFIRINOX based on the first CT-scan available after the start of therapy. One patient with R0-resection did not respond to mFOLFIRINOX and suffered a relapse five months after surgery. A therapy response was confirmed in four patients treated with gemcitabine as first-line therapy and in two patients who were treated with gemcitabine/nab-paclitaxel. One patient responded to gemcitabine monotherapy as second-line therapy after being treated with mFOLFIRINOX. In another case, the therapy was de-escalated to gemcitabine monotherapy from gemcitabine/nab-paclitaxel due to side effects. The patient had responded to gemcitabine/nab-paclitaxel but showed progression upon treatment with gemcitabine monotherapy. No differences in clinical response were observed between patients according to their TNM-status.

**Table 2:** Characteristics of patients included for organoid establishment

	Resectable disease (*n* = 8)	Metastatic disease (*n* = 5)
**Age at diagnosis** (mean)	44–84 (65)	63–79 (70)
**Sex** Male Female	3 5	2 3
**Tumor specimen** Punch biopsy Surgical resection	0 8	2 3
**R-status** R0-resection R1-resection	6 2	- -
**Sample derived from** Primary tumor Metastasis	8 -	1 5
**Weeks until therapy initation** (mean)	1–4 (2.8)	4–12 (7.1)
**First-line therapy** mFOLFIRINOX Gemcitabine/nab-paclitaxel Gemcitabine monotherapy	5 0 3	2 2 1
**Response to first-line therapy** mFOLFIRINOX Gemcitabine/nab-paclitaxel Gemcitabine monotherapy	4 - 3	2 2 1
**Second-line therapy** Gemcitabine monotherapy	1	1
**Response to second-line therapy** Gemcitabine monotherapy	1	0

### Pharmacokinetic modelling of maximum tissue concentrations (c_max/tissue_)

**Fig. 3:**
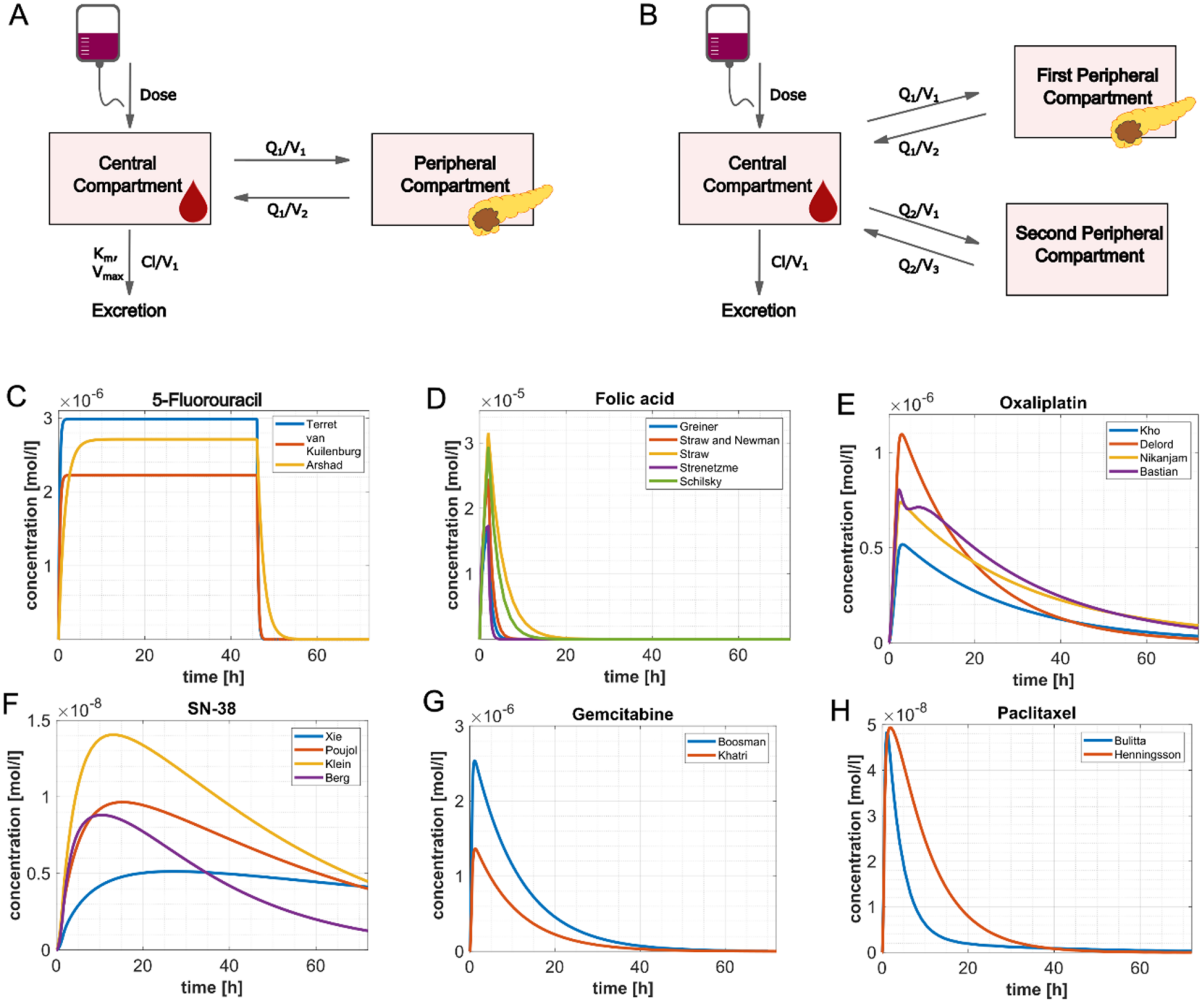
Using pharmacokinetic modelling to determine tumor tissue concentrations of chemotherapeutic agents. **A** Schematic of a two-compartment model with infusion of the drug into the central compartment (e.g. blood). Distribution into the peripheral compartment and back into the central compartment followed linear kinetics. Elimination of drugs can be modelled either with linear kinetics or with Michaelis-Menten kinetics (used for 5-Fluorouracil). **B** Schematic of a three-compartment model with infusion of the drug into the central compartment and linear elimination. Distribution into both the first and second peripheral compartment were modelled using linear kinetics. The tumor concentrations were derived from the first peripheral compartment. **C-H** Tissue concentration of oxaliplatin, SN-38, 5-FU, folic acid, gemcitabine and paclitaxel over a time span of 72 h after the start of infusion. Different lines represent tissue concentrations calculated using values from different publications. Two to five publications with pharmacokinetic values were available per drug, the first author is listed for each publication

We successfully calculated c_max/tissue_-values of oxaliplatin, SN-38, 5-FU, folic acid, gemcitabine and paclitaxel (see Table 3) based on previously reported pharmacokinetic compartment models (see Fig. 3A and B and Supplementary Table 4). When conducting a literature research to validate these concentrations, c_max/plasma_-values were within an order of magnitude of our results. Furthermore, we identified studies where the c_max/tissue_ was measured directly in the tumor for 5-FU, folic acid, gemcitabine and paclitaxel (see Supplementary Table 5). Our calculated c_max/tissue_-values were found to be within a similar range to those reported in the literature.

We identified three publications for pharmacokinetic modelling for 5-FU [59–61] all of which reported two-compartment pharmacokinetics with linear distribution [80]. 5-FU is metabolized by the enzyme dihydropyrimidin-dehydrogenase (DPD) and subsequently excreted by renal filtration [81]. Representing the enzymatic metabolization of 5-FU, all publications employed Michaelis-Menten kinetics (see Fig. 3C).

For folic acid, no parameters for multi-compartment models had previously been reported. The drug is known to distribute rapidly throughout the whole body and is therefore best modelled by a one-compartment model. Since folic acid is rapidly excreted renally, linear elimination kinetics were implemented [82]. Folic acid is known to have two isomers which are administered to patients as a racemate. Only the L-isomer has been reported to have anti-cancer effects. Pharmacokinetic modelling of c_max/tissue_ for folic acid, thus, only took into account the L-isomer concentrations (see Fig. 3D) [62–64, 66, 83].

For oxaliplatin, four studies were included for the pharmacokinetic simulation of tissue concentrations [55–58]. Two-compartment modeling was reported as the most accurate in two publications [55, 56] and two other studies used three-compartment modeling [57, 58]. We applied both two- and three compartment models to take all possible ways of the drug’s distribution into account (see Fig. 3E). Linear elimination was the most accurate for oxaliplatin known to be mainly excreted renally [84].

Irinotecan is known to be metabolized to the active metabolite SN-38 which shows an increased anti-tumoral activity by a 100 to 1000-fold factor [85]. Therefore, SN-38 was used in all experiments and its tissue concentration was calculated. Irinotecan is enzymatically converted to SN-38 by liver carboxylesterases with a conversion rate between 12.0% and 41.2% [54, 86]. In clinically relevant doses, no saturation of this enzymatic system has been observed and the metabolization was described by linear kinetics [87]. We modeled irinotecan distribution using three- and SN-38 two-compartments with linear excretion for both irinotecan and SN-38 (see Fig. 3F) [51–54].

Gemcitabine is reported to show linear distribution. The drug is rapidly deaminated by cytidine deaminase and subsequently excreted, which leads to a short plasma half-life [88]. Gemcitabine excretion occurs in two phases with an initial rapid elimination phase followed by a long terminal elimination [88]. Since these characteristics are typical for drugs with two-compartmental pharmacokinetics, we used a two-compartment model with linear distribution and excretion (see Fig. 3G) [67, 68].

Since paclitaxel is known to be the active metabolite of nab-paclitaxel, paclitaxel has been used in previously reported PDO experiments [20, 35, 36]. Paclitaxel binds to plasma proteins and blood cells in a saturable manner best described by Michaelis-Menten-kinetics. When unbound, paclitaxel can diffuse into the tissue linearly [69, 70]. Three-compartment models have been reported to be the most accurate for paclitaxel in several pharmacokinetic studies [69, 70, 89–91]. We identified two different publications for calculating paclitaxel pharmacokinetics, both studies employed linear kinetics for paclitaxel distribution and elimination (see Fig. 3H) [69, 70].

**Table 3:** Clinically administered dose and calculated values of tissue concentrations of individual chemotherapeutic agents

Drug	Dose	Infusion time	Range of c_max/tissue_	Mean of c_max/tissue_
**5-Fluorouracil [59–61]**	2400 mg/m²	46 h	2.23–2.99 µM	2.64 µM
**Folic acid [62–66]**	400 mg/m²	2 h	16.83–31.55 µM	23.9 µM
**Oxaliplatin [55–58]**	85 mg/m²	2 h	0.52–1.1 µM	0.79 µM
**SN-38 [51–54]**	150 mg/m²	1.5 h	5.13–14.06 nM	9.42 nM
**Gemcitabine [67**,** 68]**	1000 mg/m²	0.5 h	1.37–2.54 µM	1.95 µM
**Paclitaxel [69**,** 70]**	125 mg/m²	0.5 h	48.39–49.35 nM	48.87 nM

c_max/tissue_ – maximum tissue concentration, drugs are printed in bold

### Statistical clustering and Pharmacokinetic classification approaches reach high prediction accuracies

**Fig. 4:**
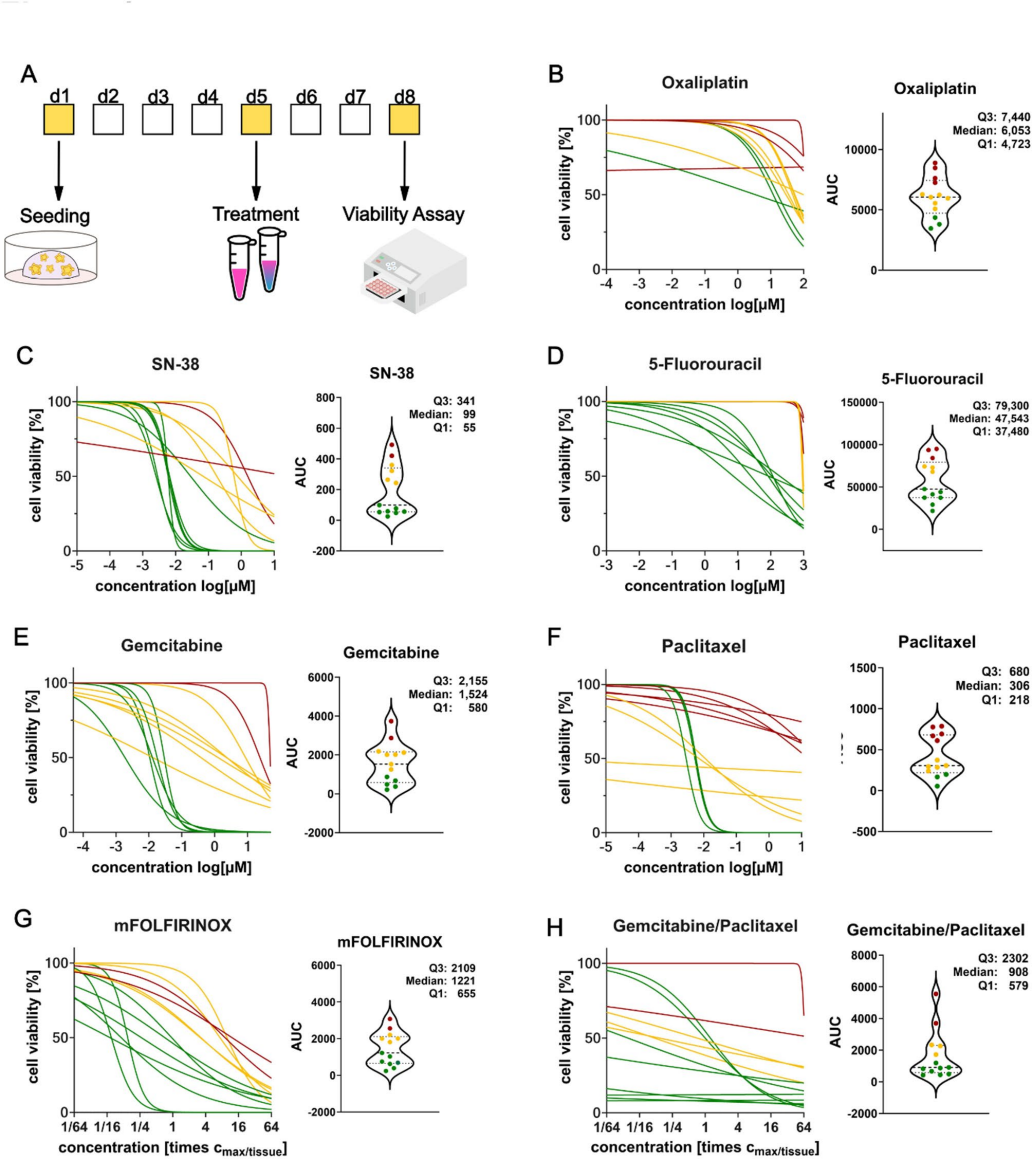
Measuring PDO drug-response towards single agents and multi-drug regimens. Clustering into sensitive, intermediary and resistant was done based on AUC values by JNB classification. **A** Experimental setup to measure cell viability in dependence of drug concentration. **B-F** Dose-response curves and violin plots of AUC values for single-agent drug tests (Oxaliplatin, SN-38, 5-Fluorouracil, Gemcitabine, Paclitaxel). **G-H** Dose-response curves and violin plots of AUC values for multi-drug tests (mFOLFIRINOX, Gemcitabine/Paclitaxel)

**Fig. 5:**
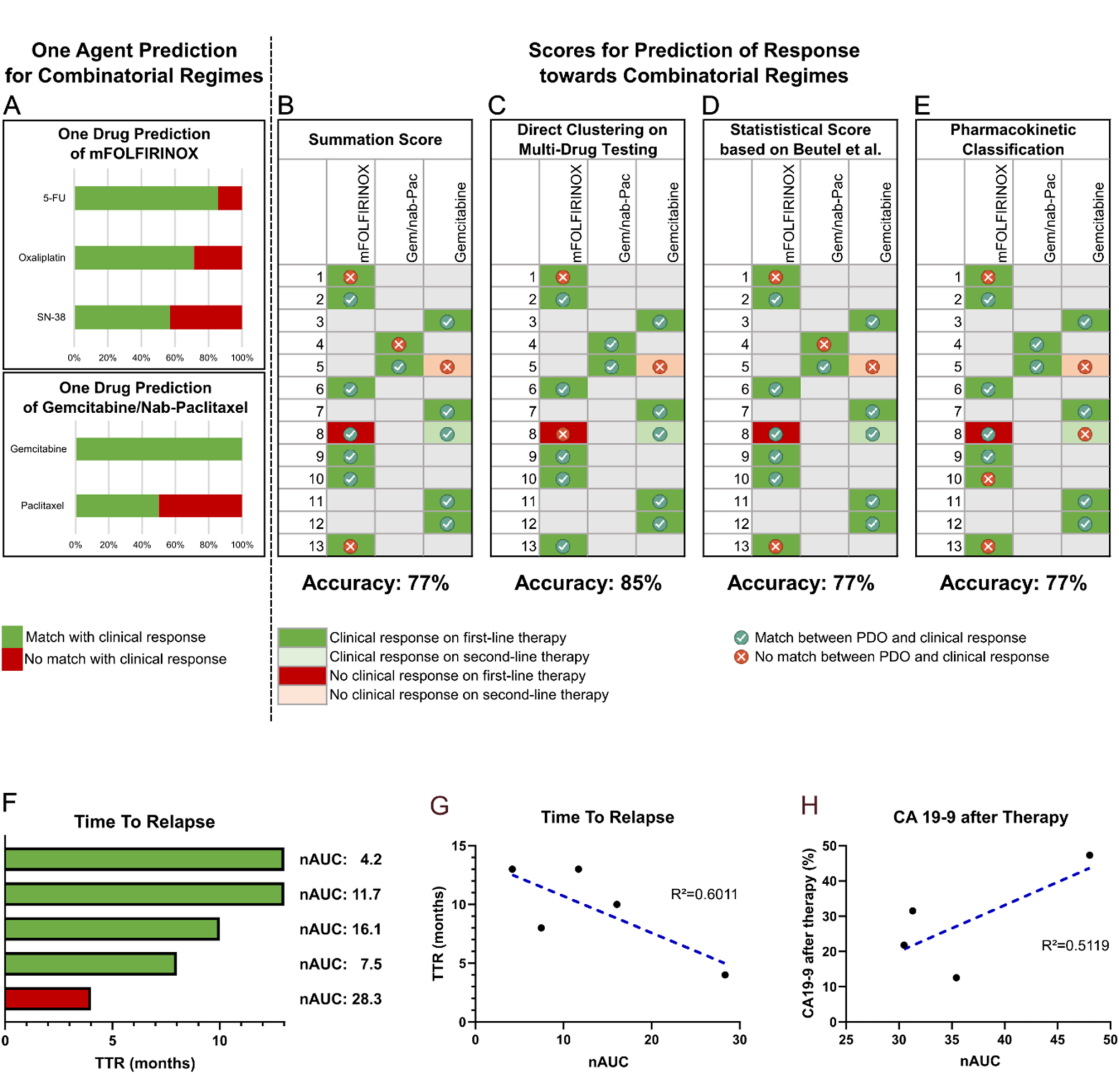
Match between in-vitro and clinical response. **A** One Agent Prediction based on AUC-values for each chemotherapeutic of mFOLFIRINOX and gemcitabine/paclitaxel, **B** AUC-based Summation Score with an accuracy of 77% for first-line therapies, **C** Direct Clustering of AUC-values of Multi-Drug Testing with an accuracy of 85% for first-line therapies, **D** Established Statistical Score for comparison of results with an accuracy of 77% and **E** the Pharmacokinetic Classification method with an accuracy of 77%. **F** Bar plot of TTR in months and corresponding nAUC, **H** TTR in months correlates with nAUC and **G** CA 19 − 9 levels after chemotherapy correlate with nAUC

In a first step, clinical response was linked to the in-vitro results of single-agent testing (see Figs. 4B-F and 5A; Table 4). Using AUC-based scores, PDO response towards oxaliplatin matched in five of seven cases (71%) and SN-38 in four of seven cases (57%). 5-FU had the highest accuracy for six out of seven patients (86%). When using log[IC_50_]-based scores, oxaliplatin and SN-38 reached higher prediction accuracy (71%) than 5-FU (57%). For gemcitabine/nab-paclitaxel, gemcitabine pharmacotyping correctly classified response using both AUC and log[IC_50_] in two of two patients (100%), while paclitaxel pharmacotyping matched with the clinical response in one of two patients (50%) for both AUC- and log[IC_50_]-values.

When using the summation score for mFOLFIRINOX therapy prediction, clinical response was correctly predicted in five of seven patients (71%) for both AUC- and log[IC_50_]-values (see Table 4). The summation score for gemcitabine/paclitaxel matched with the clinical response of patients receiving gemcitabine/nab-paclitaxel in one of two patients (50%). This was identical for both AUC- and log[IC_50_]-based scores. Overall, response was correctly classified in ten of thirteen cases for both AUC based-scores and log[IC_50_]-based scores, which corresponds to an accuracy of 77% for first-line therapies (see Figs. 4B-H and 5B; Table 5 and Supplementary Fig. 1).

**Table 4:** Match between PDO pharmacotyping and clinical response for single agent pharmacotyping

	Drugs	Accuracy AUC	Accuracy log[IC_50_]
**One agent prediction**	5-FU	6 of 7 (86%)	4 of 7 (57%)
	Oxaliplatin	5 of 7 (71%)	5 of 7 (71%)
	SN-38	4 of 7 (57%)	5 of 7 (71%)
	Gemcitabine	2 of 2 (100%)	2 of 2 (100%)
	Paclitaxel	1 of 2 (50%)	1 of 2 (50%)
**Summation Score**	mFOLFIRINOX	5 of 7 (71%)	5 of 7 (71%)
	Gem/Pac	1 of 2 (50%)	1 of 2 (50%)

When directly clustering AUC-values of multi-drug testing into sensitive, intermediary and resistant, response to first-line therapies was correctly predicted in eleven of thirteen cases, which corresponds to an accuracy of 85%, and in one of two cases for second-line therapies (see Table 5). Based on log[IC_50_]-values, response to first-line therapies was correctly predicted in eight of thirteen patients which corresponds to an accuracy of 62% (see Supplementary Fig. 1). The sensitivity of the AUC-based direct clustering approach was 92% and the specificity was 100% (see Supplementary Fig. 2). Thus, the direct clustering approach using AUC values of multi-drug testing showed the highest accuracy (see Figs. 4E, G and H and 5C).

To compare the results of our newly developed scores to established classifications, we calculated an AUC-based score as published by Beutel et al. [35]. This score correctly predicted response in ten of thirteen patients for first-line therapy, corresponding to an accuracy of 77%, and in one of two patients for second-line therapies. This previously established AUC-based classification proved to have the same prediction accuracy as the summation score described above (see Fig. 5D; Table 5).

For all statistical scores, uncertainty of accuracies was estimated using leave-one-out cross validation and bootstrapping. Leave-one-out cross validation resulted in a median match rate of 69% for Direct Clustering and the Summation Score and a median match rate of 77% for the classification established by Beutel et al. [35]. When performing bootstrapping, the median accuracy was 84.6% (CI: 54–92%) for Direct Clustering, 77% (CI: 39–100%) for the Summation Score and 77% (CI: 46–92%) for the score established by Beutel et al. [35] (see Supplementary Table 6).

**Table 5:** Overview of predictive accuracy for all statistical scores

	Summation Score		Multi-Drug Testing		Comparison to Established Score
**Drug response metric**	AUC	log[IC_50_]	AUC	log[IC_50_]	AUC
**Patients matching**	10 of 13	10 of 13	11 of 13	8 of 13	10 of 13
**Accuracy**	77%	77%	85%	62%	77%

When using the pharmacokinetic classification based on comparing IC_50_- to c_max/tissue_-values, response towards first-line therapies was correctly classified for ten of thirteen patients. This corresponds to an accuracy of 77% (see Fig. 5E). For second line therapies, the clinical response could not be correctly classified for any patient.

### Correlating in-vitro response to time to relapse and decrease in CA 19 − 9

The in-vitro response was correlated to the TTR in resected patients. TTR was available for five of eight patients in this group. Longer TTR matched with a lower nAUC in four out of five patients (R² = 0.6011, see Fig. 5F and G). For metastasized patients, the in-vitro response was correlated to the decrease in CA 19 − 9. CA 19 − 9 values were available before the start of systemic palliative therapy and two to four months after for four of five patients. Here, we observed a correlation (R² = 0.5119) between a bigger decrease in CA 19 − 9 and lower nAUC-values (see Fig. 5H).

Overall, TTR and CA 19 − 9 values were correlated with the nAUC in seven of nine patients. While this observation only includes a small number of patients, it supports the finding that AUC-values of multi-drug testing are a robust metric to determine drug response.

### Deriving classifiers from time to relapse and decrease in CA 19 − 9

Furthermore, the in-vivo data was used to derive a different in-vitro classification. TTR and CA 19 − 9 were known for five patients who received mFOLFIRINOX, three patients for gemcitabine and one patient for gemcitabine/paclitaxel. For mFOLFIRINOX, two patients were clinically classified as resistant and three as sensitive. For gemcitabine as well as gemcitabine/nab-paclitaxel, all patients were clinically classified as sensitive. Based on the clinical grouping of sensitive and resistant patients, in-vitro cut-off values of cell viability and AUCs were able to be determined for mFOLFIRINOX. Since patients treated with gemcitabine and gemcitabine/nab-paclitaxel were all identified to be clinically sensitive, no grouping of sensitive and resistant patients could be made.

The in-vitro cut-off value of cell viability was identified at the concentration that resulted in the biggest variance in cell viability between PDOs. This was identified to be 0.25*c_max/tissue_ mFOLFIRINOX. The most ideal in-vitro cut-off for cell viability was 70%. For AUC-values, the best cut-off was 1,421. At the identified cut-offs for cell viability and AUC-values, sensitive patients were identified with a sensitivity of 66.7% and a specificity of 100% (see Supplementary Fig. 3). However, using cell viabilities to identify a cut-off resulted in a lower area under the ROC-curve (0.67) than using AUC-values (0.83). Overall, the AUC remained the most reliable drug-response metric in our cohort.

### Classification of synergistic, additive and antagonistic effects in multi-drug testing

Lastly, the synergistic, additive and antagonistic effects were further analyzed in our PDO cohort. In most cases, multi-drug therapies displayed antagonistic effects (see Supplementary Fig. 4). mFOLFIRINOX was antagonistic in nine, additive in one and synergistic in three PDOs. Gemcitabine/paclitaxel showed antagonism in ten and synergy in three PDOs.

## Discussion

This study strongly supports the potential for therapy prediction of PDOs in PDAC research. Results of pharmacotyping were used to determine the most ideal way of comparing in-vitro drug response to patients’ clinical response. In contrast to exposing PDO models to single drugs, a more realistic approach is thought to expose PDOs to all drugs of each therapeutic regimen at the same time, i.e. multi-drug testing. Although this takes into account interactive effects of each drug [92–94], very few studies actually implemented multi-drug testing [48, 95–97]. Previously published studies on PDAC PDOs have focused on assessing drug response towards single agents, whereas results of multi-drug testing were correlated to the patient’s clinical response of one patient only [97]. In this work, a methodology for performing multi-drug tests for both mFOLFIRINOX and gemcitabine/nab-paclitaxel was introduced. In order to assess the potential of multi-drug testing for in-vivo therapy prediction, two different classification methods for response were implemented (direct clustering vs. pharmacokinetic). The AUC-based direct clustering of multi-drug testing performed best, reaching an accuracy of 85% for first-line therapies, which was further supported when including CA 19 − 9 and TTR as clinical response parameters. The high accuracy was also confirmed when performing bootstrapping and leave-one-out cross validation. The pharmacokinetic classification method had a prediction accuracy of 77%. These accuracies are comparable to previously performed PDO studies where response was correctly classified in 60–100% of patients (see Supplementary Table 7).

With regard to previously performed drug tests, the use of single-agent testing does not adequately reflect the clinical treatment. In the clinic, most PDAC patients are treated with combination therapies, either mFOLFIRINOX or gemcitabine/nab-paclitaxel, whereas gemcitabine monotherapy is only recommended for patients with reduced performance status [98]. For both multi-drug regimens, drug interactions have been identified in-vitro [41, 42, 99–101]. Inhibitors of topoisomerase-1, including irinotecan, increased the effect of DNA-damaging agents such as oxaliplatin by decelerating the repair of DNA-crosslinks [41]. Folic acid increased the intracellular amount of folates, which then build a complex with the enzyme thymidylate synthase and 5-FU, leading to more effective enzyme inhibition and reduced DNA replication and cell proliferation [102]. Interestingly, Di Paolo et al. [100] demonstrated that an enhanced anti-tumor effect of 5-FU combined with folic acid is only present when both drugs are administered simultaneously. Moreover, a study by Allen-Coyle et al. [101], dual and triple combinations of mFOLFIRINOX agents showed either synergistic, additive or antagonistic effects in different PDAC cell lines and culture conditions (2D vs. 3D). In this study, 5-FU and oxaliplatin showed mostly antagonistic effects, while synergistic effects occurred for the triple combination in two cell lines. Regarding the combination of gemcitabine and paclitaxel, paclitaxel has been shown to increase the concentration of gemcitabine in the tumor tissue by inhibition of the enzyme cytidine deaminase leading to reduced gemcitabine elimination [42]. In clinical studies, the benefit of multi-drug therapies can mostly be accounted to drug additivity [103, 104]. A study by Palmer et al. [103] showed that the combination of 5-FU and oxaliplatin has a significant benefit in patients with advanced PDAC over single agents, but it is unclear whether this is due to additive or synergistic effects [93, 103]. In our PDO cohort, mFOLFIRINOX and gemcitabine/paclitaxel displayed additive, synergistic and antagonistic effects. Considering the evidence of pharmacodynamic interactions both in-vivo and in-vitro as well as the results of this work, multi-drug tests are thought to be beneficial in future PDO-based translational trials. This is underlined by the high prediction accuracy of multi-drug testing in the study presented, emphasizing pharmacodynamic effects in organoid-based drug testing. So far, PDAC PDO studies used single-agent testing for therapy prediction. This clearly does not adequately reflect pharmacological interactions and does not represent the clinical treatment of patients. In order to perform multi-drug testing, the ratio of drugs were previously determined by setting drug concentrations at IC_25_- or IC_50_-values of single drugs [95, 96]. Yet, this approach is purely empirical and does not take into account in-vivo pharmacokinetic mechanisms. Peschke et al. [97] used the maximum plasma concentration (c_max/plasma_) to determine drug ratios. However, using the c_max/plasma_ does not take into account tissue distributions of drugs, which can lead to significant changes in drug concentrations. A simplified pharmacokinetic approach was employed by Bachir et al. [48]. In this study, tumor tissue concentrations (c_max/tissue_) of drugs were estimated based on an equilibrium between plasma and tissue concentrations (see Supplementary Table 8). To the best of the authors knowledge, this study is the first, employing pharmacokinetic modeling to deduce optimal concentrations for translational pharmacotyping and comparing the results to the clinical response of PDAC patients.

In our work, the pharmacokinetic classification led to lower accuracies compared with statistical approaches. This could be explained by two different limitations of the approach we presented. First of all, we were not able to verify the pharmacokinetic calculations in our patient cohort. The most ideal way to validate these calculations would have been to directly measure c_max/tissue_ in our patients. However, this was in direct contrast with our goal of obtaining therapy-naïve samples. Furthermore, biopsies during chemotherapy are not standard clinical practice. Therefore, c_max/tissue_-values remained an estimation which we verified based on literature research. Secondly, it is also important to emphasize that this classification method was based on IC_50_-values, which have previously been reported to vary significantly and to be easily affected by curve-fitting [105, 106]. Therefore, the AUC as the integral area of dose response curves is thought to be a more robust value and led to higher prediction accuracies of in-vitro drug testing [106, 107]. Another major limitation of this study remains the small patient cohort. It should be noted, however, that this patient cohort is in the range to previously published PDO cohorts [20, 35, 37, 39, 74, 108]. The majority of previously reported PDO-studies included ten to fifteen patients. Beutel et al. [35], and Tiriac et al. [20] used a larger cohort of PDOs to develop the in-vitro based statistical classification methods. However, the clinical outcome was only known for 57% and 14% of all patients respectively. Recently, a bigger study including 87 with advanced PDAC patients was published by Boilève et al. [109], where clinical correlation was feasible in 39% of cases. Another limitation of PDO studies is due to the lack of the tumor stroma. As PDOs represent only the epithelial component of a tumor, interactions with the tumor microenvironment, influencing chemotherapy resistance and drug response, cannot be captured [26, 110–112]. In the future, co-culture models can provide a more comprehensive insight into PDAC drug response [113, 114]. The generalizability of previous studies was also limited by heterogenous pharmacotyping and statistical approaches as well as a heterogenous definition of clinical response [26]. In the context of the study presented, patients with both metastatic and primary resectable disease were included. Thus, presenting a heterogeneous patient cohort.

In conclusion, the high accuracy for matching PDO-drug response to the in-vivo response of patients is in accordance to previously reported translational studies [20, 35, 37, 39, 74, 108] and strongly supports the use of PDOs as an effective platform for future translational drug testing in PDAC. Moreover, this study emphasized the need to uniformly define the way PDAC PDOs are pharmacotyped (single drug testing vs. multi-drug testing) and are further classified into sensitive and resistant models. PDOs have previously been investigated in several studies and have shown promising results for predicting patients’ treatment response. In the future, prospective clinical trials with uniform classification approaches and bigger patient cohorts will be needed to effectively translate PDO-based drug testing into clinical practice. Due to the pharmacodynamic interaction of drugs and the higher predictability of multi-drug testing, we expect future PDO studies as well as PDO-based clinical trials to use the combination of drugs.

## Conclusions

In this study, the potential of PDOs as a predictive therapy platform was highlighted and different scores for therapy prediction were evaluated using PDAC PDO models. Both statistical clustering and pharmacokinetic classification methods demonstrated good performance and reached prediction accuracies of up to 85%. This is the first study to systematically compare and contrast different classifications of in-vitro response in order to identify the score with the highest prediction accuracy. Our findings demonstrate that multi-drug testing enhances the predictive ability of PDO pharmacotyping to 85%, highlighting the importance of interactive pharmacodynamic effects. As PDOs represent a valuable platform for therapy response prediction in PDAC, the methods and results described are of high significance for future PDO-based translational trials.

## Supplementary Information

Supplementary Material 1

Corrected Tables

## Data Availability

The data included in this study is presented in this published article as well as the supplementary information.

## Abbreviations

5-FU: 5-Fluorouracil
AUC: Area under the curve
c_max/tissue_, [mmol/l]: Maximum concentration of a drug in the tumor tissue, calculated by pharmacokinetic modeling
c_max/plasma_, [mmol/l]: Maximum plasma concentration of a drug
Gem/Pac: Gemcitabine/paclitaxel
IC_50_, [mmol/l]: Half-maximal inhibitory concentration
JNB: Jenks Natural Breaks
log[IC_50_], [log[mmol/l]]: Logarithmic transformation of the half-maximal inhibitory concentration
mFOLFIRINOX: Folic acid, 5-Fluorouracil, Irinotecan and Oxaliplatin
nAUC: Normalized AUC
nlog[IC_50_], [log[mmol/l]]: Normalized log[IC_50_
PDAC: Pancreatic ductal adenocarcinoma
PDO: Patient-derived organoid
TTR: Time To Relapse
