# Supplementary material for: Multi-drug pharmacotyping improves therapy prediction in pancreatic cancer organoids
Source: Cancer Cell Int. 2025 Sep 13;25:321. doi: 10.1186/s12935-025-03969-7 (PMC12433005; doi:10.1186/s12935-025-03969-7)
Supplement: Supplementary file 1 — Supplementary Material 1 [file 12935_2025_3969_MOESM1_ESM.docx]

Supplementary

1. **Tables**

**Supplementary Table 1:** Composition of cell culture medium

| Compound | Final concentration |
| --- | --- |
| Advanced DMEM/F12 (#12634028) |  |
| Penicillin-Streptomycin (100x) | 1:100 |
| HEPES (1M) | 10 mM |
| GlutaMAX Supplement (100x) | 1:100 |
| N-Acetyl-L-Cysteine | 1 mM |
| N2-Supplement (100x) | 1:100 |
| B27-Supplement (50x) | 1:50 |
| Wnt-3a conditioned medium | 30% (vol/vol) |
| R-Spondin I conditioned medium | 5% (vol/vol) |
| Nicotinamide (#N0636-100G/500G) | 10 mM |
| Gastrin I | 10 nM |
| EGF (#E9644-.2MG) | 50 ng/ml |
| FGF10 (#100-26) | 100 ng/ml |
| A 83-01 (#2939) | 500 nM |
| Noggin (#120-10C) | 100 ng/ml |
| Y27632 2HCl (#M1817) | 10 µM |

**Supplementary Table 2:** Variables used for pharmacokinetic modelling

| Variable | Unit | Explanation |
| --- | --- | --- |
| D | mg | Amount of drug infused |
| T | h | Time of infusion |
| x_1_ | mg | Amount of drug in central compartment |
| x_2_ | mg | Amount of drug in first peripheral compartment |
| x_3_ | mg | Amount of drug in second peripheral compartment |
| V_1_ | l | Volume of central compartment |
| V_2_ | l | Volume of first peripheral compartment |
| V_3_ | l | Volume of second peripheral compartment |
| Q_1_ | l/h | Intercompartmental Clearance between central and first peripheral compartment |
| Q_2_ | l/h | Intercompartmental Clearance between central and second peripheral compartment |
| Cl | l/h | Clearance from central compartment |
| e | mg | Amount of drug excreted |
| V_max_ | mg/h | Maximum speed of elimination |
| K_m_ | mg/l | Michaelis-Menten Constant |

**Supplementary Table 3:** Overview of established vs. non-established PDOs

|  | Established (N=25) | No growth (N=18) | p-Value |
| --- | --- | --- | --- |
| Sample type |  |  | 0.111 |
| Punch Biopsy | 4 (16.0%) | 0 (0%) |  |
| Surgical Sample | 21 (84.0%) | 17 (94.4%) |  |
| Fine Needle Biopsy | 0 (0%) | 1 (5.6%) |  |
| Sample Size |  |  | 0.086 |
| Median (Min – Max) | 0.615 (0.125 - 5.52) | 1.19 (0.125 - 6.00) |  |
| Sample weight |  |  | 0.771 |
| Median (Min – Max) | 0.459 (0.0369 - 2.74) | 0.830 (0.0460 - 1.32) |  |
| Age |  |  | 0.578 |
| Median (Min - Max) | 66.0 (44.0 - 86.0) | 69.5 (54.0 - 91.0) |  |
| Sex |  |  | 0.476 |
| Female | 19 (76.0%) | 11 (61.1%) |  |
| Male | 6 (24.0%) | 7 (38.9%) |  |
| UICC-Stage |  |  | 0.306 |
| I | 4 (16.0%) | 4 (22.2%) |  |
| II | 9 (36.0%) | 8 (44.4%) |  |
| III | 3 (12.0%) | 4 (22.2%) |  |
| IV | 9 (36.0%) | 2 (11.1%) |  |
| T-Stage |  |  | 0.683 |
| T1 | 2 (12.5%) | 1 (6.3%) |  |
| T2 | 10 (62.5%) | 9 (56.3%) |  |
| T3 | 4 (25.0%) | 5 (31.3%) |  |
| T4 | 0 (0%) | 1 (6.3%) |  |
| N-Stage |  |  | 0.71 |
| N0 | 4 (25.0%) | 6 (37.5%) |  |
| N1 | 8 (50.0%) | 6 (37.5%) |  |
| Grading |  |  | 0.386 |
| G1 | 0 (0%) | 1 (6.3%) |  |
| G2 | 9 (56.3%) | 7 (43.8%) |  |
| G3 | 5 (31.3%) | 3 (18.8%) |  |
| Gx | 2 (12.5%) | 5 (31.3%) |  |

**Supplementary Table 4:** Pharmacokinetic models used for calculation of tissue concentrations

| Chemotherapeutic | Number of compartments | Distribution kinetic | Elimination kinetic |
| --- | --- | --- | --- |
| 5-Flurorouracil | 2 | Linear | Saturable |
| Folic acid | 1 | - | Linear |
| Oxaliplatin | 2 and 3 | Linear | Linear |
| SN-38 | 3 for irinotecan  2 for SN-38 | Linear | Linear |
| Gemcitabine | 2 | Linear | Linear |
| Paclitaxel | 3 | Linear | Linear |

**Supplementary Table 5:** C_max/plasma_- and C_max/tissue_-values of cytostatics identified by literature research. C_max/plasma_-values were chosen only for drug concentrations applied in PDAC treatment, whereas C_max/tissue_-values were accepted for different dosage regimes since there was less available data.

|  | **Values identified by literature research** | | **Values calculated by pharmacokinetic modeling** | |
| --- | --- | --- | --- | --- |
|  | **c_max/plasma_** | **c_max/tissue_** | **c_max/plasma_** | **c_max/tissue_** |
| **5-FU** | 7.5 µM [1] | 0.4 – 1.23 µM [2] | 2.63 µM | 2.64 µM |
| **Folic acid** | 1.85 µM [3]  18.8 µM [4] | 71.7 µM [5] | 23.9 µM | 23.9 µM |
| **Oxaliplatin** | 2.5 µM [6]  3.6 µM [7] |  | 3.508 µM | 0.79 µM |
| **SN-38** | 74 nM [8] |  | 57.09 nM | 9.42 nM |
| **Gemcitabine** | 17 µM [9] | 3.48µM [10, 11] | 39.43 µM | 1.95 µM |
| **Paclitaxel** | 5 µM [12] | 940 nM [13]  500 nM [14] | 380.3 nM | 48.87 nM |

**Supplementary Table 6:** Results of Bootstrapping for Direct Clustering, the Summation Score and the Score by Beutel et al.

|  | **Mean Accuracy** | **Standard Error** | **95% Confidence Interval** |
| --- | --- | --- | --- |
| **Direct Clustering on Multi-Drug Testing** | 0.846 | 0.107 | 0.538 – 0.923 |
| **Summation Score** | 0.769 | 0.104 | 0.385 – 0.846 |
| **Beutel et al.** | 0.769 | 0.108 | 0.462 – 0.923 |

**Supplementary Table 7:** Previously performed PDO studies and correlation rates

| **Publication** | **Classification of response in-vitro** | **Classification of response in-vivo** | **Number of correlated cases** | **Successful correlation** |
| --- | --- | --- | --- | --- |
| **Beutel et al. [15]** | Statistical classification (Jenks Natural Breaks) | RECIST 1.1 at the first scan after start of therapy | In 16 cases | 91.1% for first-line therapies, 80% for second-line therapies |
| **Driehuis et al. [16]** | Statistical classification | Physician’s assessment of clinical response | In 4 cases | Correlation in all cases |
| **Tiriac et al. [17]** | Statistical (upper/lower/middle third) | Progression-Free Survival | In 9 cases | Correlation in all cases |
| **Grossman et al. [18]** | Statistical (JNB) | RECIST 1.1 at the first scan after start of therapy | In 12 cases | Correlation in all cases |
| **Boilève et al. [19]** | Score including the AUC and AOC, the top 25% of the score was defined as sensitivity | RECIST 1.1, Progression-Free Survival | In 34 cases | 91.2% |
| **Demyan et al. [20]** | Statistical based on Tiriac et al. | Progression-Free Survival, CA19-9 and tumor-size decrease | In 15 cases | 100% for treatment-naïve PDOs, 71% for pre-treated PDOs |
| **Farshadi et al. [21]** | Statistical | Clinical response to neoadjuvant therapy | In 5 cases | 60% |

**Supplementary Table 8:** Overview of in-vitro approaches toward combination drug therapy for PDAC

| Publication | Regimen | Combination based on | Maximum concentrations of chemotherapeutic agents |
| --- | --- | --- | --- |
| Peschke et al. [22] | FOLFIRINOX | c_max/plasma_ | 16.9 µM Irinotecan  37.6 µM 5-FU  7.9 µM Oxaliplatin |
| Bachir et al. [23] | FOLFIRINOX | Pharmacokinetic equilibrium to estimate c_max/tissue_ | 12.5 nM SN-38  4 µM 5-FU  0.5 µM Oxaliplatin |
| Hennig et al. [24] | FOLFIRINOX and gemcitabine/nab-paclitaxel | IC_50_ values | 10 μM Irinotecan  35 μM Oxaliplatin  35 μM 5-FU  14 nM Gemcitabine  9 nM Paclitaxel |
| Farshadi et al. [21] | FOLFIRINOX | IC_25_ values | 5nM SN-38  30 µM 5-FU  10 µM Oxaliplatin |

1. **Figures**

**
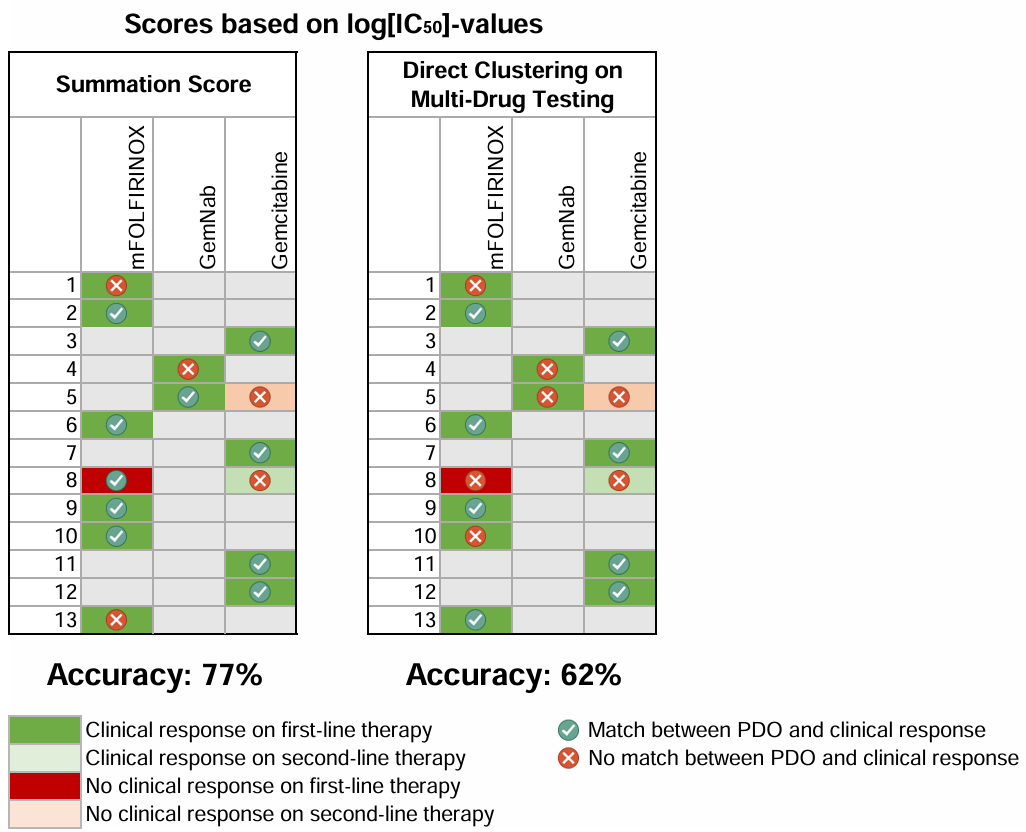
**

**Supplementary Figure 1:** Overview of log[IC_50_]-based scores


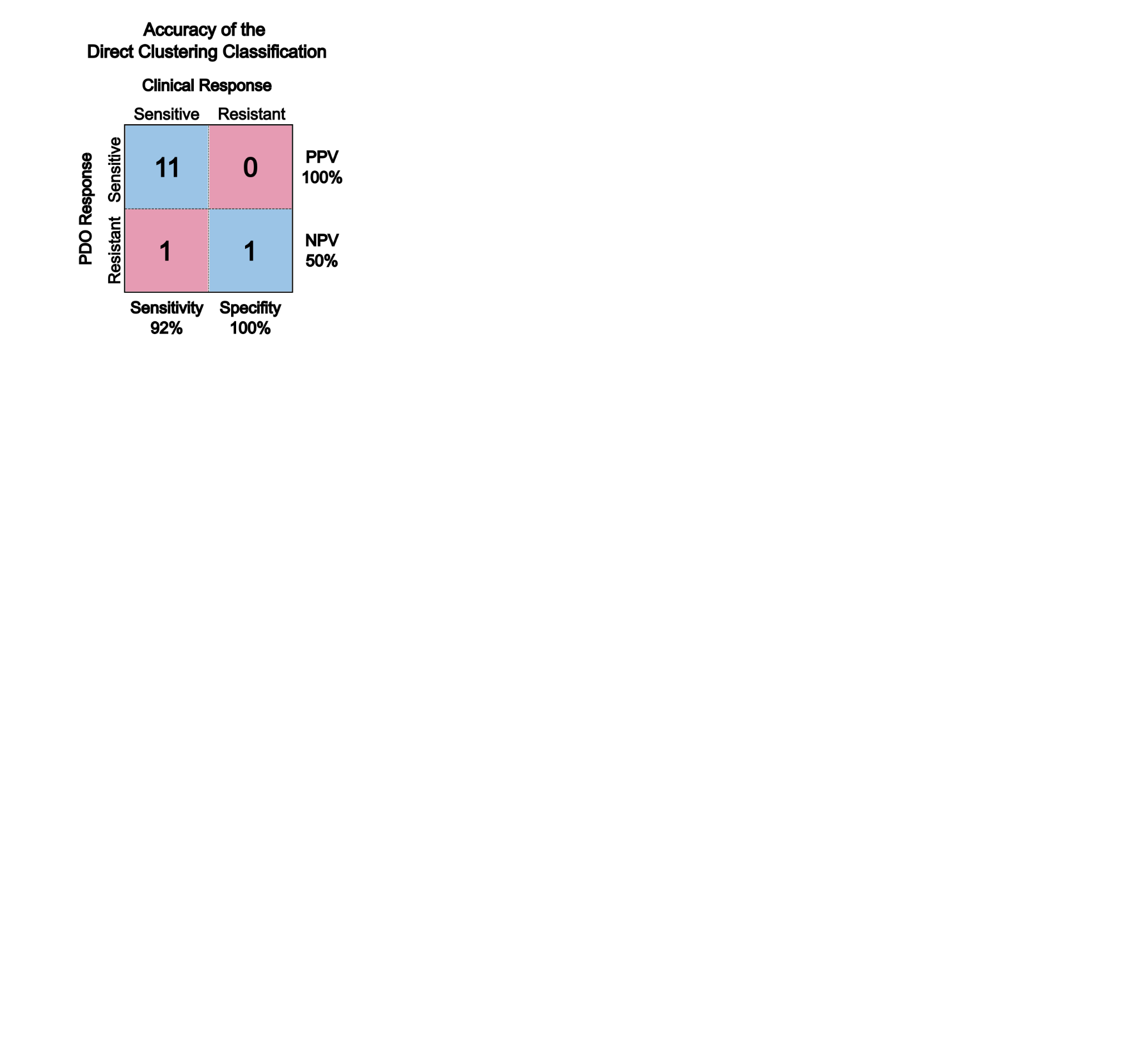


**Supplementary Figure 2**: 2x2-table including specificity, sensitivity, PPV and NPV of the Direct Clustering Classification

*
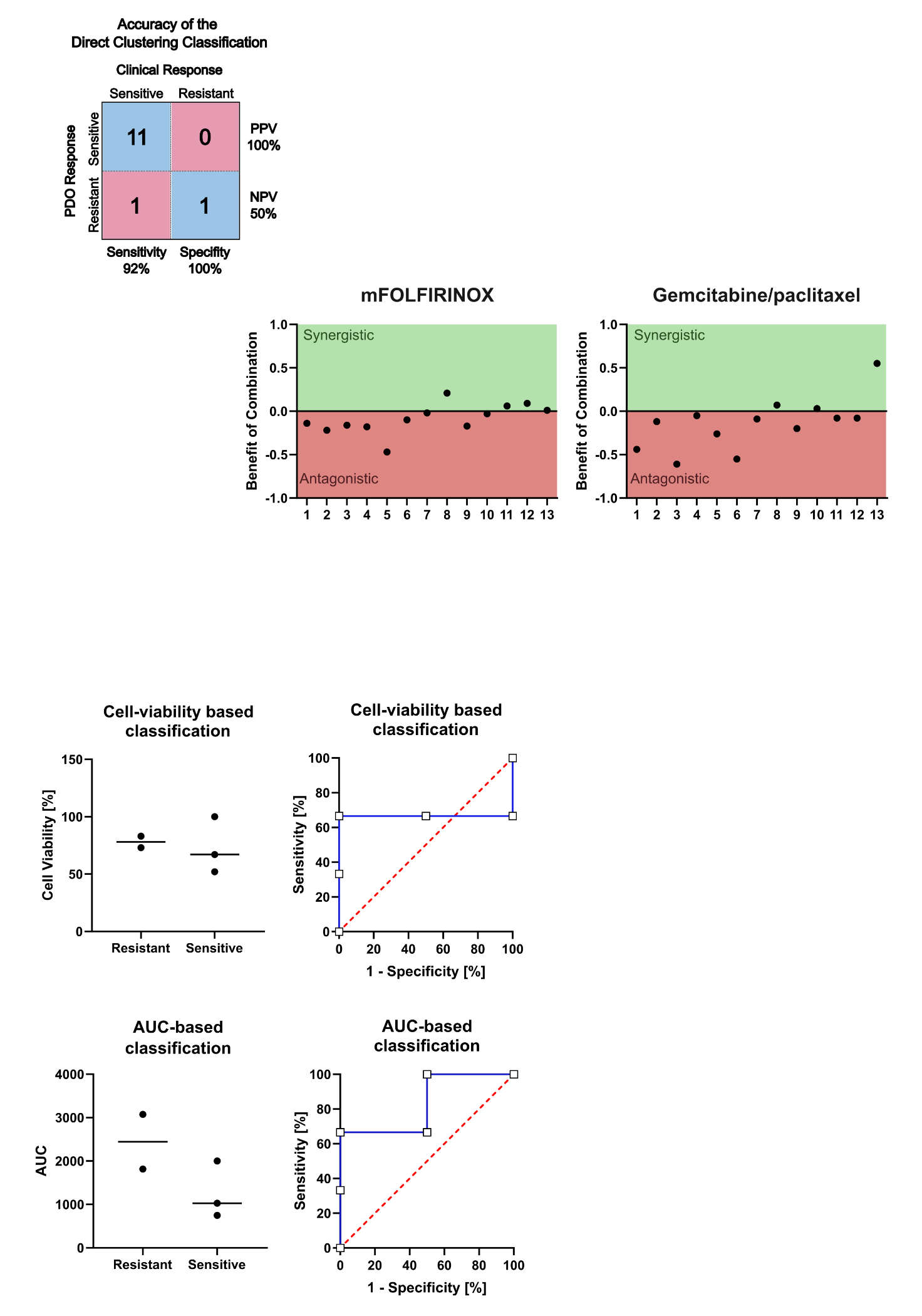
*

**Supplementary Figure 3:** Cell-viability based classification and AUC-based classification of mFOLFIRINOX response. Patients with a decrease in CA 19-9 below 60% and a TTR shorter than 6 months were classified as resistant, those with greater decrease and longer TTR were classified as sensitive.


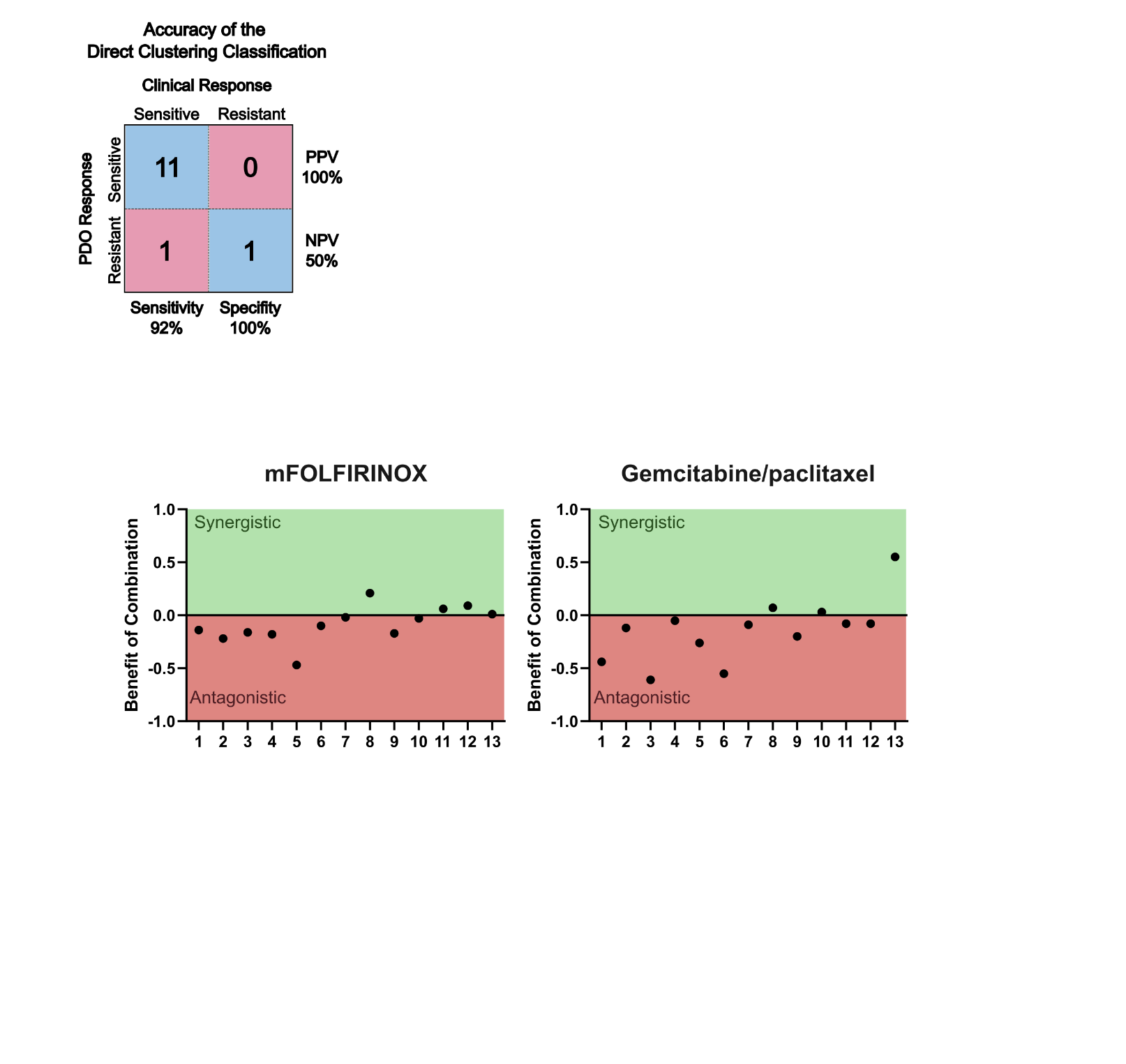


**Supplementary Figure 4**: Synergistic and antagonistic effects of combination therapy in PDO models. On the Y-axis, the additional decrease in cell viability with combinational treatment is displayed. An additional value of 0% equals additivity, values below 0 equal antagonism, values above 0 equal synergy.

**References:**

1. Blaschke M, Blumberg J, Wegner U, Nischwitz M, Ramadori G, Cameron S: **Measurements of 5-FU Plasma Concentrations in Patients with Gastrointestinal Cancer: 5-FU Levels Reflect the 5-FU Dose Applied**. *Journal of Cancer Therapy* 2012, **3**:1.

2. Konings IR, Sleijfer S, Mathijssen RH, de Bruijn P, Ghobadi Moghaddam-Helmantel IM, van Dam LM, Wiemer EA, Verweij J, Loos WJ: **Increasing tumoral 5-fluorouracil concentrations during a 5-day continuous infusion: a microdialysis study**. *Cancer Chemother Pharmacol* 2011, **67**:5.

3. Priest DG, Schmitz JC, Bunni MA, Stuart RK: **Pharmacokinetics of leucovorin metabolites in human plasma as a function of dose administered orally and intravenously**. *J Natl Cancer Inst* 1991, **83**:24.

4. Meropol NJ, Petrelli NJ, Rustum YM, Rodriguez-Bigas M, Blumenson LE, Frank C, Berghorn E, Creaven PJ: **A phase II and pharmacokinetic study of 6S-leucovorin plus 5-fluorouracil in patient with colorectal carcinoma**. *Invest New Drugs* 1995, **13**:2.

5. Straw JA, Newman EM: **Pharmacokinetic Analysis of (6S)-5-Formyltetrahydrofolate (1-CF), (6R)-5-Formyltetrahydrofolate (d-CF) and 5-Methyltetrahydrofolate (5-CH3-THF) in Patients Receiving Constant i.v. Infusion of High-Dose (6R,S)-5-Formyltetrahydrofolate (Leucovorin)**. In: *The Expanding Role of Folates and Fluoropyrimidines in Cancer Chemotherapy Advances in Experimental Medicine and Biology.* vol. 244. New York, NY: Springer; 1988.

6. Ehrsson H, Wallin I, Yachnin J: **Pharmacokinetics of oxaliplatin in humans**. *Medical Oncology* 2002, **19**:4.

7. Kho Y, Jansman FGA, Prins NH, Neef C, Brouwers JRBJ: **Population Pharmacokinetics of Oxaliplatin (85 mg/m2) in Combination With 5-fluorouracil in Patients With Advanced Colorectal Cancer**. *Therapeutic Drug Monitoring* 2006, **28**:2.

8. Rothenberg ML, Eckardt JR, Kuhn JG, 3rd HAB, Nelson J, Hilsenbeck SG, Rodriguez GI, Thurman AM, Smith LS, Eckhardt SG *et al*: **Phase II trial of irinotecan in patients with progressive or rapidly recurrent colorectal cancer**. *Journal of Clinical Oncology* 1996, **14**:4.

9. Alharbi AF, Kratzke RA, D’Cunha J, Maddaus MA, Sanghavi K, Kirstein MN: **Gemcitabine and metabolite pharmacokinetics in advanced NSCLC patients after bronchial artery infusion and intravenous infusion**. *Cancer Chemotherapy and Pharmacology* 2019, **83**:2.

10. Green AL, Flannery P, Hankinson TC, O’Neill B, Amani V, DeSisto J, Knox A, Chatwin H, Lemma R, Hoffman LM *et al*: **Preclinical and clinical investigation of intratumoral chemotherapy pharmacokinetics in DIPG using gemcitabine**. *Neuro-Oncology Advances* 2020, **2**:1.

11. Burris HA, 3rd, Moore MJ, Andersen J, Green MR, Rothenberg ML, Modiano MR, Cripps MC, Portenoy RK, Storniolo AM, Tarassoff P *et al*: **Improvements in survival and clinical benefit with gemcitabine as first-line therapy for patients with advanced pancreas cancer: a randomized trial**. *J Clin Oncol* 1997, **15**:6.

12. Stage TB, Bergmann TK, Kroetz DL: **Clinical Pharmacokinetics of Paclitaxel Monotherapy: An Updated Literature Review**. *Clinical Pharmacokinetics* 2018, **57**:1.

13. Heimans JJ, Vermorken JB, Wolbers JG, Eeltink CM, Meijer OW, Taphoorn MJ, Beijnen JH: **Paclitaxel (Taxol) concentrations in brain tumor tissue**. *Ann Oncol* 1994, **5**:10.

14. Koshiba H, Hosokawa K, Mori T, Kubo A, Watanabe Ai, Honjo H: **Intravenous Paclitaxel Is Specifically Retained in Human Gynecologic Carcinoma Tissues In Vivo**. *International Journal of Gynecological Cancer* 2009, **19**:4.

15. Beutel AK, Schutte L, Scheible J, Roger E, Muller M, Perkhofer L, Kestler A, Kraus JM, Kestler HA, Barth TFE *et al*: **A Prospective Feasibility Trial to Challenge Patient-Derived Pancreatic Cancer Organoids in Predicting Treatment Response**. *Cancers (Basel)* 2021, **13**:11.

16. Driehuis E, van Hoeck A, Moore K, Kolders S, Francies HE, Gulersonmez MC, Stigter ECA, Burgering B, Geurts V, Gracanin A *et al*: **Pancreatic cancer organoids recapitulate disease and allow personalized drug screening**. *Proc Natl Acad Sci U S A* 2019, **116**:52.

17. Tiriac H, Belleau P, Engle DD, Plenker D, Deschenes A, Somerville TDD, Froeling FEM, Burkhart RA, Denroche RE, Jang GH *et al*: **Organoid Profiling Identifies Common Responders to Chemotherapy in Pancreatic Cancer**. *Cancer Discov* 2018, **8**:9.

18. Grossman JE, Muthuswamy L, Huang L, Akshinthala D, Perea S, Gonzalez RS, Tsai LL, Cohen J, Bockorny B, Bullock AJ *et al*: **Organoid Sensitivity Correlates with Therapeutic Response in Patients with Pancreatic Cancer**. *Clin Cancer Res* 2022, **28**:4.

19. Boilève A, Cartry J, Goudarzi N, Bedja S, Mathieu JRR, Bani M-A, Nicolle R, Mouawia A, Bouyakoub R, Nicotra C *et al*: **Organoids for Functional Precision Medicine in Advanced Pancreatic Cancer**. *Gastroenterology* 2024, **167**:5.

20. Demyan L, Habowski AN, Plenker D, King DA, Standring OJ, Tsang C, St. Surin L, Rishi A, Crawford JM, Boyd J *et al*: **Pancreatic Cancer Patient-derived Organoids Can Predict Response to Neoadjuvant Chemotherapy**. *Annals of Surgery* 2022, **276**:3.

21. Farshadi EA, Chang J, Sampadi B, Doukas M, Van 't Land F, van der Sijde F, Vietsch EE, Pothof J, Koerkamp BG, van Eijck CHJ: **Organoids Derived from Neoadjuvant FOLFIRINOX Patients Recapitulate Therapy Resistance in Pancreatic Ductal Adenocarcinoma**. *Clinical Cancer Research* 2021, **27**:23.

22. Peschke K, Jakubowsky H, Schafer A, Maurer C, Lange S, Orben F, Bernad R, Harder FN, Eiber M, Ollinger R *et al*: **Identification of treatment-induced vulnerabilities in pancreatic cancer patients using functional model systems**. *EMBO Mol Med* 2022, **14**:4.

23. Hadj Bachir E, Poiraud C, Paget S, Stoup N, El Moghrabi S, Duchene B, Jouy N, Bongiovanni A, Tardivel M, Weiswald LB *et al*: **A new pancreatic adenocarcinoma-derived organoid model of acquired chemoresistance to FOLFIRINOX: First insight of the underlying mechanisms**. *Biol Cell* 2022, **114**:1.

24. Hennig A, Baenke F, Klimova A, Drukewitz S, Jahnke B, Brückmann S, Secci R, Winter C, Schmäche T, Seidlitz T *et al*: **Detecting drug resistance in pancreatic cancer organoids guides optimized chemotherapy treatment**. *The Journal of Pathology* 2022, **257**:5.
