# Supplementary material for: Multi-drug pharmacotyping improves therapy prediction in pancreatic cancer organoids
Source: Cancer Cell Int. 2025 Sep 13;25:321. doi: 10.1186/s12935-025-03969-7 (PMC12433005; doi:10.1186/s12935-025-03969-7)
Supplement: Supplementary file 2 — Corrected Tables [file 12935_2025_3969_MOESM2_ESM.docx]

**Table 2:** Characteristics of patients included for organoid establishment

|  | Resectable disease (*n* = 8) | Metastatic disease (*n* = 5) |
| --- | --- | --- |
| Age at diagnosis (mean) | 44-84 (65) | 63-79 (70) |
| Sex  Male  Female | 3  5 | 2  3 |
| Tumor specimen  Punch biopsy  Surgical resection | 0  8 | 2  3 |
| R-status  R0-resection  R1-resection | 6  2 | -  - |
| Sample derived from  Primary tumor  Metastasis | 8  - | 1  5 |
| Weeks until therapy initation (mean) | 1-4 (2.8) | 4-12 (7.1) |
| First-line therapy  mFOLFIRINOX  Gemcitabine/nab-paclitaxel  Gemcitabine monotherapy | 5  0  3 | 2  2  1 |
| Response to first-line therapy  mFOLFIRINOX  Gemcitabine/nab-paclitaxel  Gemcitabine monotherapy | 4  -  3 | 2  2  1 |
| Second-line therapy  Gemcitabine monotherapy | 1 | 1 |
| Response to second-line therapy  Gemcitabine monotherapy | 1 | 0 |
| Headings are printed in bold | | |

**Table 3:** Clinically administered dose and calculated values of tissue concentrations of individual chemotherapeutic agents

| Drug | Dose | Infusion time | Range of c_max/tissue_ | Mean of c_max/tissue_ |
| --- | --- | --- | --- | --- |
| 5-Fluorouracil [59-61] | 2400 mg/m² | 46 h | 2.23 – 2.99 µM | 2.64 µM |
| Folic acid [62-66] | 400 mg/m² | 2 h | 16.83 – 31.55 µM | 23.9 µM |
| Oxaliplatin [55-58] | 85 mg/m² | 2 h | 0.52 – 1.1 µM | 0.79 µM |
| SN-38 [51-54] | 150 mg/m² | 1.5 h | 5.13 – 14.06 nM | 9.42 nM |
| Gemcitabine [67, 68] | 1000 mg/m² | 0.5 h | 1.37 – 2.54 µM | 1.95 µM |
| Paclitaxel [69, 70] | 125 mg/m² | 0.5 h | 48.39 – 49.35 nM | 48.87 nM |
| c_max/tissue_ – maximum tissue concentration, drugs are printed in bold | | | | |

**Table 4:** Match between PDO pharmacotyping and clinical response for single agent pharmacotyping.

|  | Drugs | Accuracy AUC | Accuracy log[IC_50_] |
| --- | --- | --- | --- |
| One agent prediction | 5-FU | 6 of 7 (86 %) | 4 of 7 (57 %) |
|  | Oxaliplatin | 5 of 7 (71 %) | 5 of 7 (71 %) |
|  | SN-38 | 4 of 7 (57 %) | 5 of 7 (71 %) |
|  | Gemcitabine | 2 of 2 (100 %) | 2 of 2 (100 %) |
|  | Paclitaxel | 1 of 2 (50 %) | 1 of 2 (50 %) |
| Summation Score | mFOLFIRINOX | 5 of 7 (71 %) | 5 of 7 (71 %) |
|  | Gem/Pac | 1 of 2 (50 %) | 1 of 2 (50 %) |
| Scores used for classification are printed in bold | | | |
